# The *Trichinella* Super‐Pangenome Reveals the Evolution of Encapsulation and Predicted Host–Parasite Protein Interactions

**DOI:** 10.1002/advs.202523161

**Published:** 2026-04-10

**Authors:** Qingbo Lv, Yi Liu, Ning Xu, Xiao Zhang, Yaming Yang, Chengyao Li, Hanhai Mao, Mingyuan Liu, Xiaolei Liu

**Affiliations:** ^1^ State Key Laboratory For Diagnosis and Treatment of Severe Zoonotic Infectious Diseases Institute of Zoonosis and College of Veterinary Medicine Key Laboratory for Zoonosis Research of the Ministry of Education Jilin University Changchun China; ^2^ Yunnan Institute of Parasitic Diseases Yunnan China; ^3^ China Conservation and Research Center For the Giant Panda Key Laboratory of SFGA On the Giant panda Chengdu Sichuan China; ^4^ Jiangsu Co‐innovation Center For Prevention and Control of Important Animal Infectious Diseases and Zoonoses Yangzhou China

**Keywords:** antagonistic coevolution, drug target, host–parasite interactions, pangenome, Trichinella

## Abstract

The muscle capsule of *Trichinella* is a critical structure that impedes immune attacks and drug penetration, yet the molecular mechanisms underlying its formation remain poorly understood. Using a high‐quality super‐pangenome comprising 12 *Trichinella* species, we compared extensive genomic variations between encapsulating and non‐encapsulating lineages. Pangenomic analysis revealed an open‐genome architecture dominated by dispensable genes, with nonencapsulated lineages exhibiting a high load of structural variation (SV). These findings suggest that the capsule represents an evolutionary innovation that arose in the common ancestor of *Trichinella* as an adaptation to mammalian hosts, dating back approximately 18 million years. Branch‐specific selection analysis further revealed distinct host adaptation strategies between the two lineages. By integrating cross‐species and cross‐developmental transcriptomic atlases, we constructed a secreted protein‐host interaction network and identified encapsulation‐associated parasite‐secreted proteins. Additionally, we identified *dpy‐31* as a promising drug target. Collectively, our work establishes a comprehensive genomic and functional framework that deepens the understanding of host–parasite interactions and opens avenues for therapeutic intervention.

## Graphical abstract

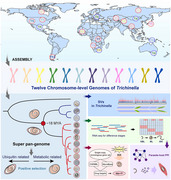

## 1 Introduction

Antagonistic coevolution is a ubiquitous phenomenon in host–parasite relationships [1, 2]. Parasitic helminths often modulate and suppress host immune responses to establish chronic infections, while hosts, in turn, develop adaptive protective mechanisms to minimize pathology. In parasitic helminths, long‐term infection is often mediated by parasite excretory‐secretory (ES) products that modulate host cellular and immune processes [3]. Despite extensive documentation of such interactions, the evolutionary and molecular mechanisms by which these strategies arise and diversify remain incompletely understood.

*Trichinella* is an obligate internal parasitic nematode whose life cycle encysts mostly within host skeletal muscle and is devoid of an external environmental phase [4]. This intimate and continuous host interaction subject the parasite to intense evolutionary pressure, making it an excellent model for studying host–parasite coevolution and immune coadaptation. A defining clinical feature of *Trichinella* infection is the formation of a collagenous capsule surrounding the nurse cell (NC) larva complex in skeletal muscle [4]. This multicellular structure, comprising the cyst wall, NCs, myoblasts, recruited monocytes, and larva, acts as a robust physical and chemical barrier that impedes drug penetration and host immune effectors, a key factor contributing to the chronic nature of trichinellosis [5]. Within the genus, three species (*T. pseudospiralis*, *T. papuae*, and *T. zimbabwensis*) that primarily infect avian/reptilian hosts exhibit attenuated or absent capsule formation and are thus classified as nonencapsulated lineages [6]. Importantly, the capsule originates from the transdifferentiation of host muscle cells rather than being a direct product of the larva, representing a quintessential outcome of host–parasite antagonistic coevolution [7]. Despite its importance, the host–parasite interactions governing capsule formation remain poorly understood.

Advances in genome sequencing have facilitated the in silico prediction of host–parasite molecular interactions. The strategy of transferring known protein interactions across species on the basis of conserved homologs has been successfully applied to interactome models of several important parasites [8, 9]. However, such approaches have remained limited in *Trichinella* due to the lack of high‐quality genome assemblies. These microscopic organisms often require pooling of numerous individuals to obtain sufficient DNA for long‐read sequencing, a process that introduces substantial heterozygosity from genetic diversity among individuals, thereby complicating de novo genome assembly.

In this study, we generated chromosome‐level genome assemblies for 12 *Trichinella* species by establishing highly inbred laboratory lines, enabling the construction of the first super‐pangenome of the genus. Using these resources, we systematically analyzed genomic diversity and evolutionary divergence between encapsulated and nonencapsulated lineages. By integrating cross‐species and cross‐stage transcriptomic data, we further predicted candidate host–parasite protein interactions and identified conserved drug targets, providing a genomic framework for investigating capsule evolution and host adaptation in *Trichinella*. Our work provides foundational genomic resources and functional insights into the molecular mechanisms of capsule formation, advancing our understanding of host–parasite antagonistic coevolution and informing future strategies for antiparasitic drug development.

## 2 Results

### 2.1 Genome Sequencing and Assembly of the Trichinella Complex

**FIGURE 1:**
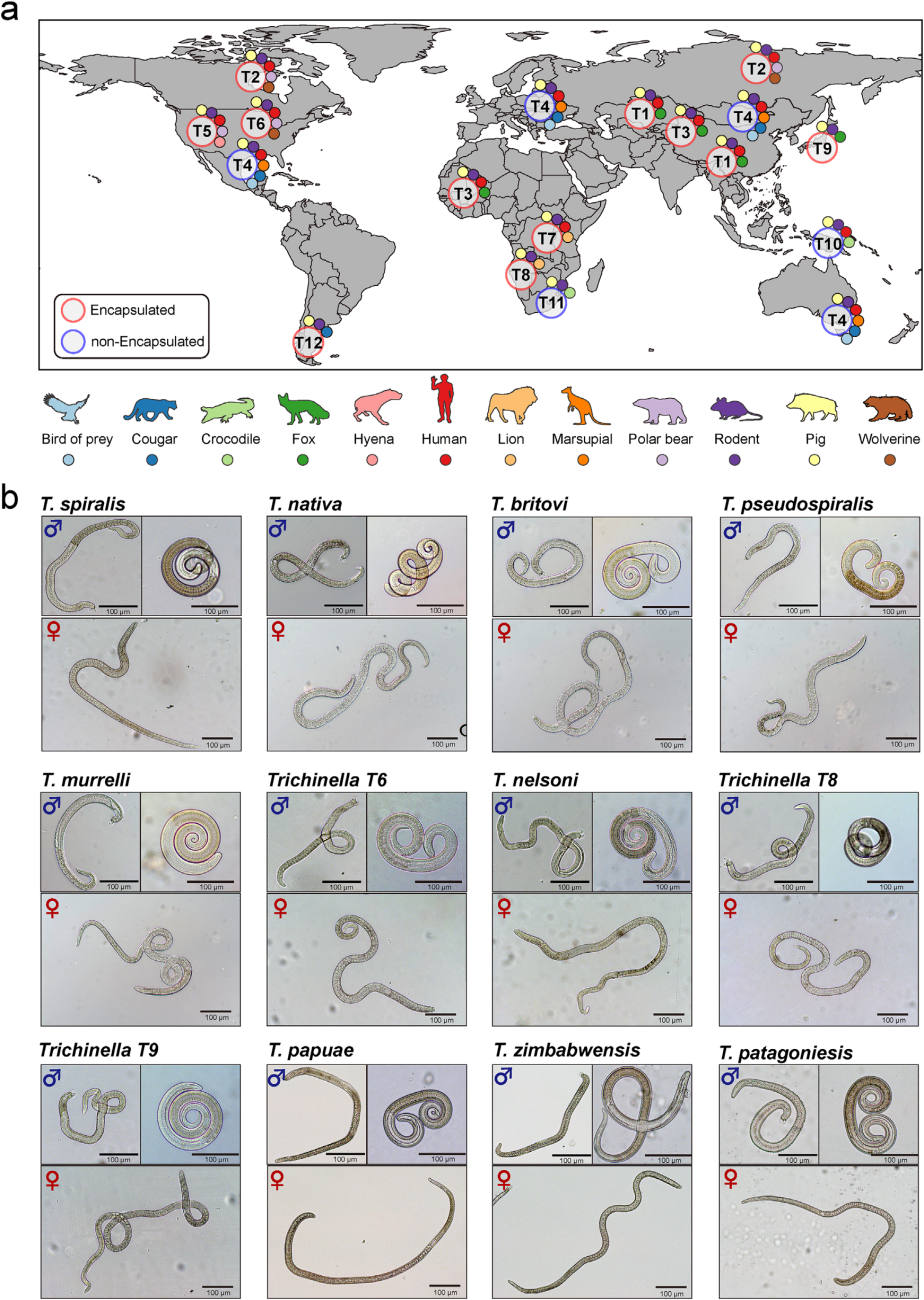
Global distribution, host diversity, and developmental morphology of *Trichinella* species. (**a)** Geographical distribution of 12 *Trichinella* species, categorized into two groups on the basis of their encapsulation phenotype: red (encapsulated) and blue (nonencapsulated). Rings on the map indicate endemic regions for each species, with surrounding‐colored dots representing common host types. (**b)** Representative images of *Trichinella* nematodes, illustrating the morphological features of muscle larvae and adults (both sexes).

The genus *Trichinella* represents an ancient nematode lineage that diverged from other nematodes approximately two hundred million years ago [7, 10]. Through adaptive radiation occurring between 20 and 3 million years ago, this genus differentiated into multiple species adaptive to different host organisms [11], subsequently achieving a nearly global distribution (Figure 1a). Our study encompasses 12 representative species (Figure 1b), constituting a comprehensive sample that includes nine encapsulated species and all currently recognized nonencapsulated species, thereby covering nearly all known members of this genus.

**FIGURE 2:**
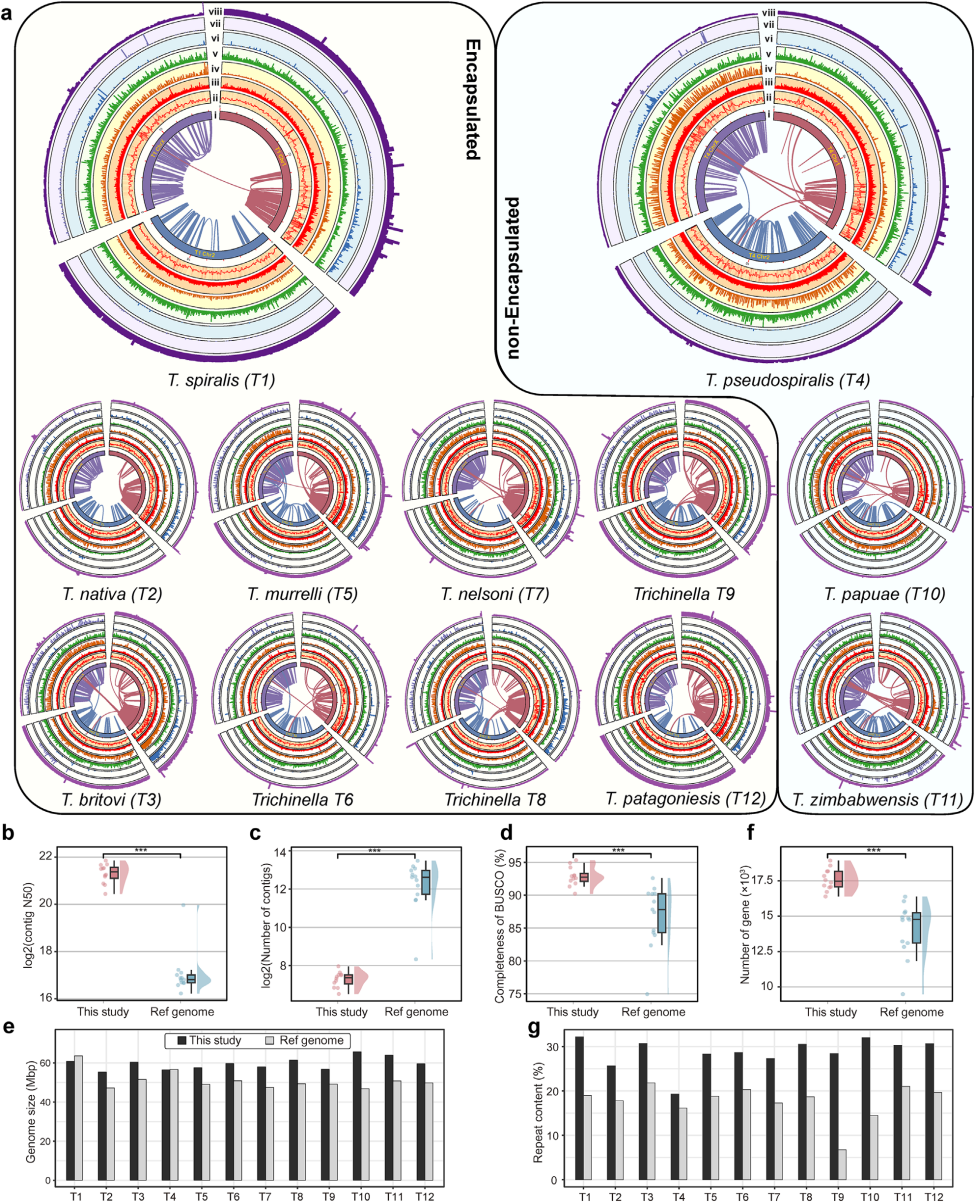
Genomic characteristics of *Trichinella* species. (**a)** Features of 12 genome assemblies, with expanded views of two representative species (T1, encapsulated; T4, nonencapsulated). Circos plot tracks (from inner to outer): (i) three assembled chromosomes; (ii) gene density; (iii) GC content; (iv) DNA repeat density; (v) simple sequence repeat density; (vi) LTR retrotransposon density; (vii) LINE/SINE density; (viii) sequencing depth of male individual resequencing data. (**b–g)** Comparative metrics between our assemblies and the current reference genomes: (b) contig N50 length, (c) number of contigs, (d) BUSCO completeness scores, (e) genome size comparison between assemblies and their corresponding reference genomes, (f) predicted gene count, and (g) repetitive sequence content comparison between assemblies and reference genomes.

A comprehensive de novo assembly strategy was employed to enhance the genome assembly of the *Trichinella* complex [*T. spiralis* (T1), *T. nativa* (T2), *T. britovi* (T3), *T. pseudospiralis* (T4), *T. murrelli* (T5), *Trichinella* T6, *T. nelsoni* (T7), *Trichinella* T8, *Trichinella* T9, *T. papuae* (T10), *T. zimbabwensis* (T11) and *T. patagoniensis* (T12)]. This strategy integrated reads from PacBio long reads (>150‐fold coverage) and Illumina paired‐end reads (>100‐fold coverage) that were generated from the muscle larva mix pool. A total of 239.26 G of data were generated using this strategy (Table S1), with an average of 9.97 G of data per *Trichinella* species. For all the species, we further used the Hi‐C approach for construction at the chromosome level and obtained a total of 79.73 G of data (>100‐fold coverage). Ultimately, we generated chromosome‐level genome assemblies for 12 *Trichinella* species (Figure 2a). These genome sizes ranged from 55.30 to 65.71 Mb (average of 59.61 Mb), the contig number ranged from 91 to 248 (average of 160.25), the scaffold N50 ranged from 16.57 to 18.65 Mb (average of 17.15 Mb), the GC content ranged from 32.43% to 34.08%, and the Hi‐C chromosome mounting rates ranged from 85.15% to 96.17% (average of 90.93%) (Table 1; Figure S1). Hi‐C data also indicated that all the *Trichinella* species had three chromosomes. The putative X chromosome for each species was determined on the basis of the coverage of the resequencing of male samples (Table S2). To assess the quality of the new assembly, we compared the new assembly with the current reference genome for each species. Compared with the original assembly, the newly assembled genome was less fragmented, exhibiting a more than 30‐fold increase in N50 length and a more than 72% reduction in scaffolds (Figure 2b,c; Table S3). The integrity of each genome supported by BUSCO scores was superior to that of previously published genomes of these species (Figure 2d; Table S3). Most of the newly assembled genomes were larger in size than the original assembly (Figure 2e). Notably, the newly assembled T1 genome (60.8 Mb) was slightly smaller in size than the current reference genome (63.5 Mb). This difference may have been due to the excessive number of gaps in the T1 reference genome (GCF_000181795.1), which contains approximately 5 Mb of gap sequences [12]. Together, these results indicated that our assembly was of better quality and had higher confidence.

**TABLE 1:** Statistics of de novo assemblies of different *Trichinella* species.

Description	T1 (ISS534)	T2 (ISS70)	T3 (ISS100)	T4 (ISS141)	T5 (ISS35)	T6 (ISS34)	T7 (ISS37)	T8 (ISS124)	T9 (ISS408)	T10 (ISS1980)	T11 (ISS1029)	T12 (ISS1826)
Country of origin	China	European	Palaearctic	China	Nearctic	America	Africa	Africa	Japan	Papua	Zimbabwe	Argentina
Host of origin	Domestic pig	Polar bear	Red fox	Birds	Coyote	Grizzly bear	Warthog	Lion	Raccoon dog	Human	Nile crocodile	Cougar
Genome size (bp)	60,778,089	55,298,063	60,369,333	56,435,383	57,473,486	59,673,582	57,950,719	61,300,507	56,740,686	65,713,553	63,918,001	59,646,474
Number of chromosomes	3	3	3	3	3	3	3	3	3	3	3	3
Number of scaffolds; contigs	171; 248	26; 113	65; 185	89; 170	65; 179	98; 137	61; 181	74; 147	58; 91	152; 199	102; 159	77; 114
GC content (%)	33.39	33.74	34.08	32.43	33.68	33.83	33.95	33.85	33.94	32.72	32.91	34
Contig N50 (bp)	2,459,534	1,854,148	2,499,384	1,422,550	3,389,984	3,009,559	3,338,753	1,925,347	2,974,216	2,370,903	3,040,425	3,793,377
Scaffold N50 (bp)	16,569,897	17,189,547	16,836,887	17,051,193	16,737,568	16,832,652	16,665,011	17,320,444	16,725,136	18,553,640	18,651,777	16,676,971
Sequence anchored to chromosomes (%)	86.24	96.17	92.92	91.84	92.67	87.58	91.44	92.26	93.56	85.15	90.19	91.13
BUSCO eukaryota (%)	92.6	92.1	92.6	90.6	92.6	92.5	91.7	92.9	92.6	92.2	90.6	92.6
BUSCO nematode (%)	70.1	71	71	70.2	70.7	70.9	70.7	70.8	70.9	70.5	70.2	70.7

We predicted the gene models for each species using ab initio, homology‐based, and transcriptome evidence‐based strategies. We obtained reliable gene structure annotations by using previously published RNA sequencing data from different developmental stages [newborn larvae (NBL), muscle larvae (ML), and adult worms (AW)] of each species [13]. All the genomic structures were supported by the RNA sequencing data (Figure S2), which indicated that the new assembly captured a more complete gene sequence (Figure 2f). Afterward, we annotated the genomes of all the species in detail. The repeat sequence annotation revealed that the proportion of repeats in these species was between 19.25% and 32.55% (average 28.67%) (Figure 2g), whereas the proportion of interspersed repeats was between 15.57% and 29.49% (Table S4). On average, each genome contained 14.08 miRNAs, 30.33 snRNAs, and 106.67 rRNAs (Table S5). We predicted an average of 17,590 gene models per species, and the number of genes ranged from 16,394 to 18,941; the average length was 2,353 bp, and the average coding sequence (CDS) length was 930 bp (Table 2). We performed functional annotation of the gene sets for all the species in public databases. More than 90% of the genes could be matched to homologous genes in the NR and InterPro databases. On average, 37.95% of the genes contained at least one Pfam entry, and 38.29% of the genes had homologous connections to one or more known Kyoto Encyclopedia of Genes and Genomes (KEGG) biological pathways (Table S6). The BUSCO evaluation results indicated that the completeness of the new gene structure annotation was significantly greater than that of the reference genomes (Table S2). These results showed that our strategy achieved more reliable genomic annotation quality.

**TABLE 2:** Details of the identification of gene structure of different *Trichinella* species.

Description	T1 (ISS534)	T2 (ISS70)	T3 (ISS100)	T4 (ISS141)	T5 (ISS35)	T6 (ISS34)	T7 (ISS37)	T8 (ISS124)	T9 (ISS408)	T10 (ISS1980)	T11 (ISS1029)	T12 (ISS1826)
Number of gene models	17,036	16,712	18,941	17,087	17,385	17,850	16,394	18,584	17,170	17,558	18,183	18,174
Number of exon	98,349	96,086	102,060	98,217	97,244	99,806	95,507	100,909	95,117	98,269	98,718	99,864
Mean gene length (bp)	2,342	2,441	2,310	2,378	2,321	2,366	2,545	2,316	2,327	2,311	2,257	2,319
Mean CDS length (bp)	928	957	896	908	930	965	996	904	935	923	902	912
Mean exon length (bp)	177	183	183	175	182	188	188	183	185	183	185	181
BUSCO eukaryota (%)	93	92.5	92.6	91.8	91.3	93	92.9	90.2	92.2	94.2	95.3	94.9
BUSCO nematode (%)	70	69.8	69.6	69.8	70.3	69.8	68.5	69.4	69.9	70.8	70.4	70.2

### 2.2 Genome Organization and Phylogeny of the *Trichinella* Genus

**FIGURE 3:**
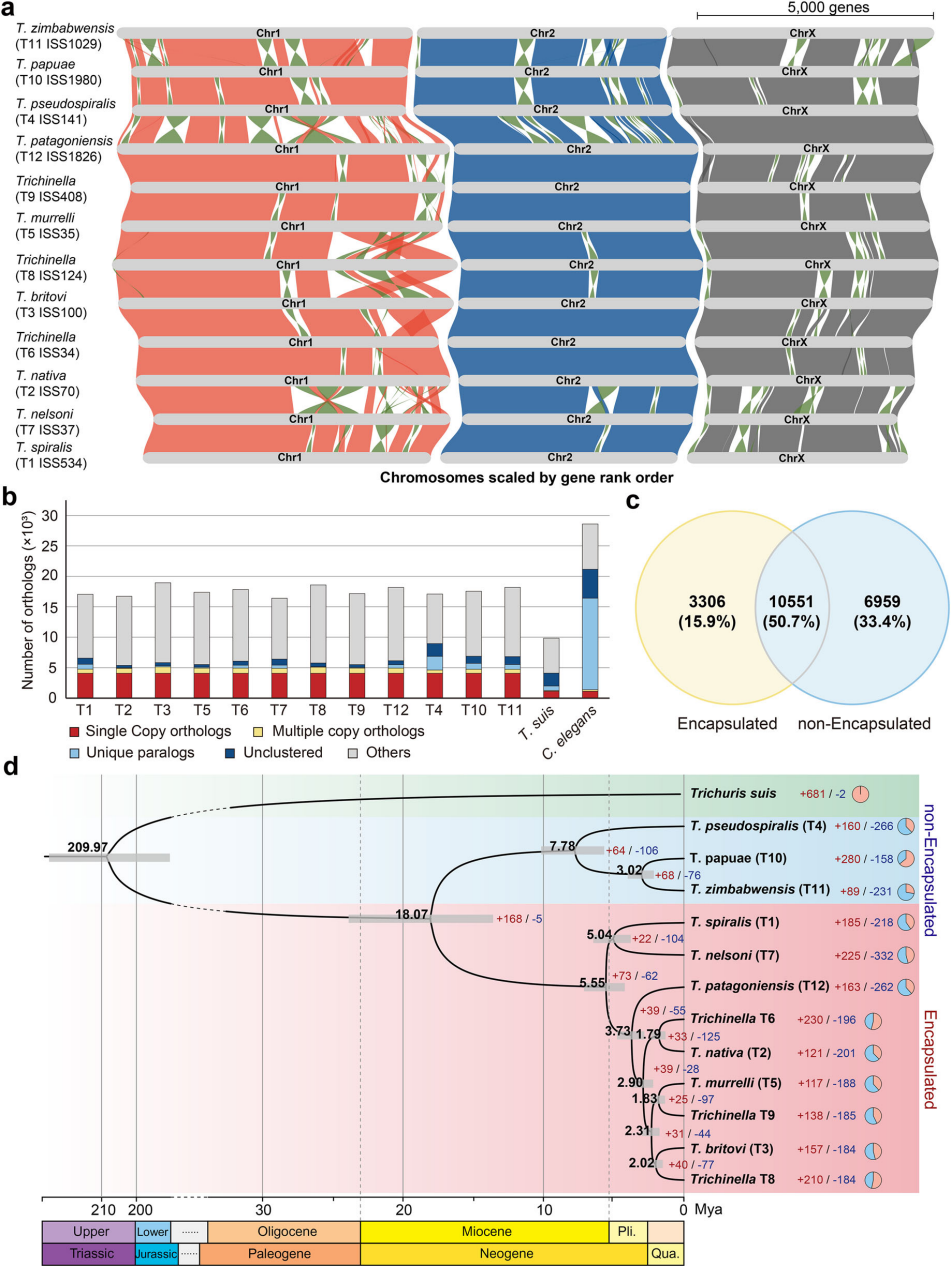
Evolutionary genomics of *Trichinella*. **(a)** Synteny analysis of *Trichinella* genomes. GENESPACE plot showing homologous regions across species, vertically arranged by the phylogenetic relationship. Large InDels and inversions (green) are evident across all species. (**b)** Gene family analysis of *Trichinella* with two outgroups (*T. suis* and *C. elegans*). Categories include single‐copy orthologs, multicopy orthologs (gene families with multiple copies in all species), others (present in ≥1 species), unique paralogs (≥2 copies without orthologs in other species), and unclustered (single‐copy genes without orthologs). (**c)** Venn diagram of shared and lineage‐specific gene families between encapsulated (red) and nonencapsulated (blue) lineages. **(d)** Phylogenetic tree representing the divergence time in *Trichinella*, with *T. suis* as the outgroup. Red/blue shaded areas denote encapsulated (9 species) and nonencapsulated (3 species) clades, respectively. Node numbers indicate estimated divergence times (Mya), with gray bars representing 95% confidence intervals. Post‐node values indicate gene family expansion (+) or contraction (−).

A synteny analysis of the genome revealed differences in the genomic structure among the *Trichinella* species. These results revealed extensive recombination of many chromosomal segments among the 12 species (Figure 3a). Chromosomal structural variations (SVs) between encapsulated and nonencapsulated species were pronounced. For example, a collinear analysis of T4 and T1 (Figure S3) revealed several large inversions on chromosome 1 and fewer rearrangements on chromosome X.

Gene family analysis revealed notable variations in orthologs among *Trichinella* species. A total of 31,117 gene families were identified. A total of 4077 gene families were single‐copy orthologs (Figure 3b, Table S7). Each species had an average of 858 unclustered genes and an average of 585 unique paralogs. Comparative profiling between encapsulated and nonencapsulated lineages revealed disproportionate gene family diversification. Remarkably, although the nonencapsulated lineages included only three members, they monopolized 33.4% of the species‐specific gene clusters (Figure 3c).

To better determine the phylogenetic relationships of *Trichinella*, we reconstructed the phylogeny of *Trichinella* groups with one outgroup species (*Trichuris suis*) on the basis of a single‐copy ortholog set (Figure 3d). Consistent with expectations, the phylogenetic tree revealed two major clades, representing encapsulated and nonencapsulated lineages. Afterward, on the basis of the differentiation time nodes (202 million years ago, Mya) of the ancestors of *T. spiralis* and *T. suis* predicted by recent studies [10], the differentiation time among *Trichinella* species was estimated. Molecular dating estimates suggested that the encapsulated and nonencapsulated *Trichinella* lineages diverged from their last common ancestor approximately 18.07 Mya (95% HPD: 13.26–23.56) (Figure 3d). The divergence between *T. spiralis* and *T. nelsoni* was calculated to have occurred approximately 5.04 Mya (3.78–6.45), while their separation from other encapsulated species was estimated to have occurred 5.55 Mya (4.21–7.11). For the nonencapsulated clade, the estimated divergence time of the most recent common ancestor was 7.78 Mya (5.67–10.17). Computational analysis of the evolution of gene families revealed widespread evolutionary contraction in species lineages (Figure 3d). Most of the species exhibited a higher rate of gene family contraction than expansion events did (Table S8). This represented the loss of redundant functions, which might be related to host adaptation and niche specialization processes.

### 2.3 Core and Dispensable Genomes

**FIGURE 4:**
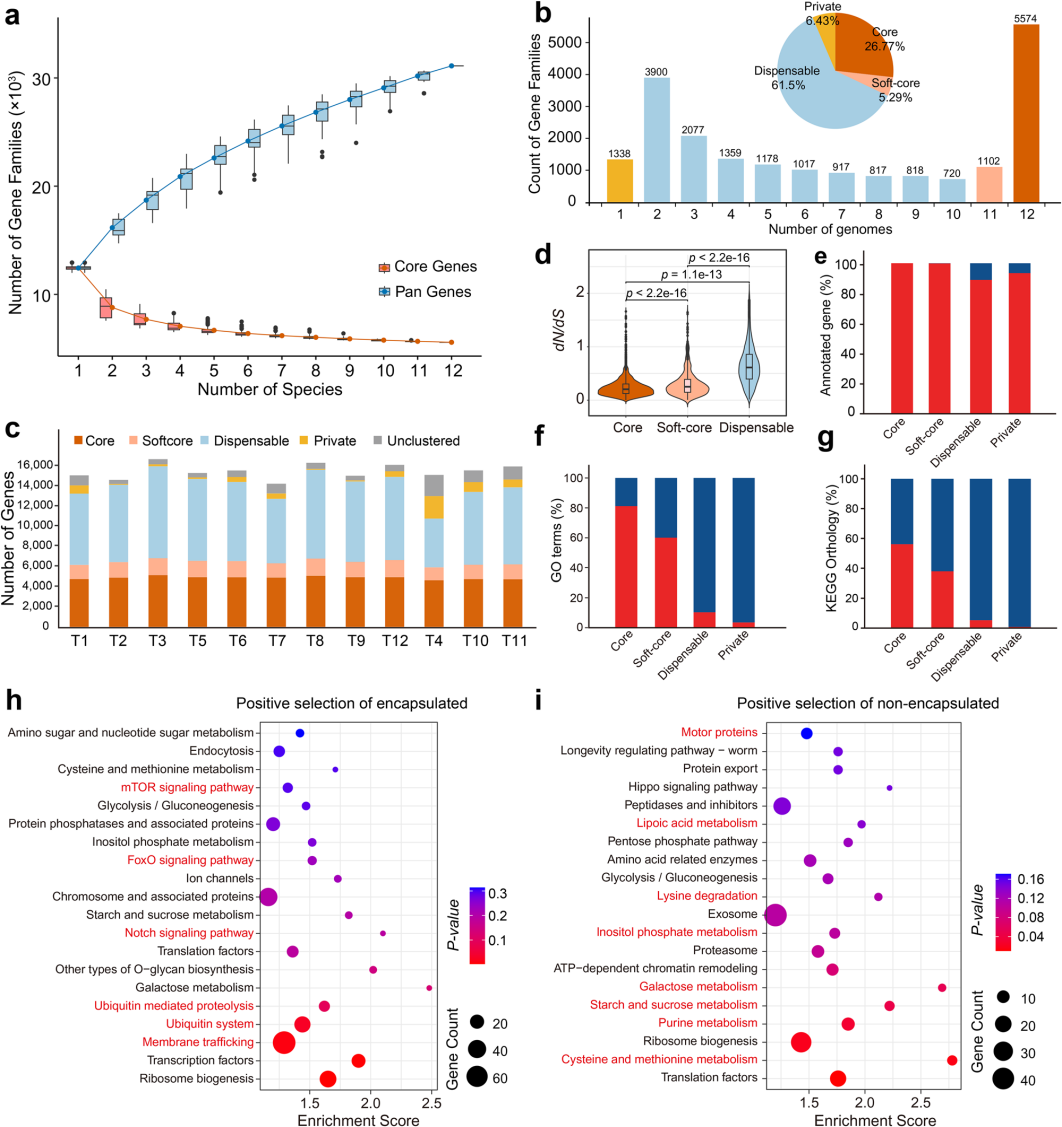
Super‐pangenome landscape of *Trichinella*. **(a)** Core and pangenome accumulation curves based on 1000 resampling iterations with increasing species number. Solid lines represent the median gene family counts. (**b)** Gene family frequency distribution. The histogram shows the counts of gene families at different frequencies across the 12 genomes; the pie chart displays the proportions of each gene family type. (**c)** Counts of each gene family type per individual genome. (**d)** d*N*/d*S* ratio comparison among core genes, soft‐core genes, and dispensable genes. (**e–g)** Functional annotation statistics for each gene family type: (e) overall functional annotation, (f) Gene Ontology (GO) terms, and (g) Kyoto Encyclopedia of Genes and Genomes (KEGG) pathways. Red/blue portions indicate annotated/unannotated gene families, respectively. (h,i) Positively selected KEGG pathways enriched in (h) encapsulated and (i) nonencapsulated lineages.

To systematically investigate Sthe genomic diversity across *Trichinella*, we constructed a super‐pangenome encompassing 12 representative species. Through comparative analysis of 211,074 predicted genes clustered into 20,817 orthologous gene families (Table S9), we identified distinct evolutionary patterns in the genome architecture across the genus. Pangenome curve analysis revealed critical insights into the genomic openness of the genus. The total number of gene families in the pangenome increased with the addition of another genome, suggesting a greater representation of individuals from the *Trichinella* genus (Figure 4a). In contrast, the core gene family decay curve stabilized at 5574 gene families, representing the conserved functional core across the genus.

The *Trichinella* pangenome exhibited a characteristic open structure dominated by accessory components, with conserved elements (core and soft‐core gene families) representing only 32.06% of the total gene families (Figure 4b). Specifically, 5574 gene families (26.77%) were classified as core gene families (universally present across all species), complemented by 1102 soft‐core gene families (5.29%, present in ≥11 species). Notably, the majority of the gene families (67.94%) were dispensable, including 12,803 dispensable gene families (61.50%) distributed across 2–10 species and 1338 private gene families (6.43%) (Figure 4b). Additionally, an average of 858 unclustered genes were identified per species, with nearly half (44.30%, 4563 genes) originating from three nonencapsulated species (Table S10). On average, 46.41% of the genes in each genome were classified as dispensable or species specific (Figure 4c), which underscores the diversity of the *Trichinella* genome. In addition, the *ω* (dN/dS) ratios of the core genes were lower than those of the soft‐core and dispensable genes (Figure 4d).

Furthermore, we conducted comparative analyses of shared gene content between phylogenetically closely related species and species‐genotype pairs. Compared with interspecies comparisons, species‐genotype comparisons revealed an approximately 10% greater number of shared genes (Figure S4), suggesting incomplete genetic differentiation between genotypes that had not yet reached full speciation. The maintained genetic continuity further supported the current taxonomic classification of these genotypes as intraspecific variants rather than distinct species.

Functional annotation revealed that 100% of the core genes, 99.9% of the soft‐core genes, and 88.9% of the dispensable genes contained at least one conserved domain (Figure 4e). Compared with dispensable genes, core and soft core genes had higher Gene Ontology (GO) and KEGG annotation rates (Figures 4f,g). The results of functional enrichment analyses indicated that the core gene family was involved in basic biological functions, such as transmembrane transport (GO:0055085), DNA‐binding transcription factor activity (GO:0000981), protein kinase activity (GO:0004672), ribosome biogenesis (ko03009), and membrane trafficking (ko04131) (Figure S5, Table S11). In contrast, dispensable gene families were enriched in functions related to DNA integration (GO:0015074), deoxyribonuclease II activity (GO:0004531), the lysosome (ko04142), the secretion system (ko02044), and folate biosynthesis (ko00790), among others (Figure S6, Table S12).

Positive selection analysis revealed the functional preferences of encapsulated and nonencapsulated lineages. This procedure resulted in the identification of 819 positively selected genes (PSGs) in encapsulated lineages versus 976 PSGs in nonencapsulated lineages (Table S13). Functional enrichment analysis revealed significant differences: the PSGs of the encapsulated lineages were predominantly associated with the membrane trafficking ubiquitin system and Notch signaling pathways, whereas the PSGs of the nonencapsulated lineages were clustered in the categories of cysteine and methionine metabolism and purine metabolism (Figure 4h,i).

### 2.4 Variation Catalog for Trichinella

**FIGURE 5:**
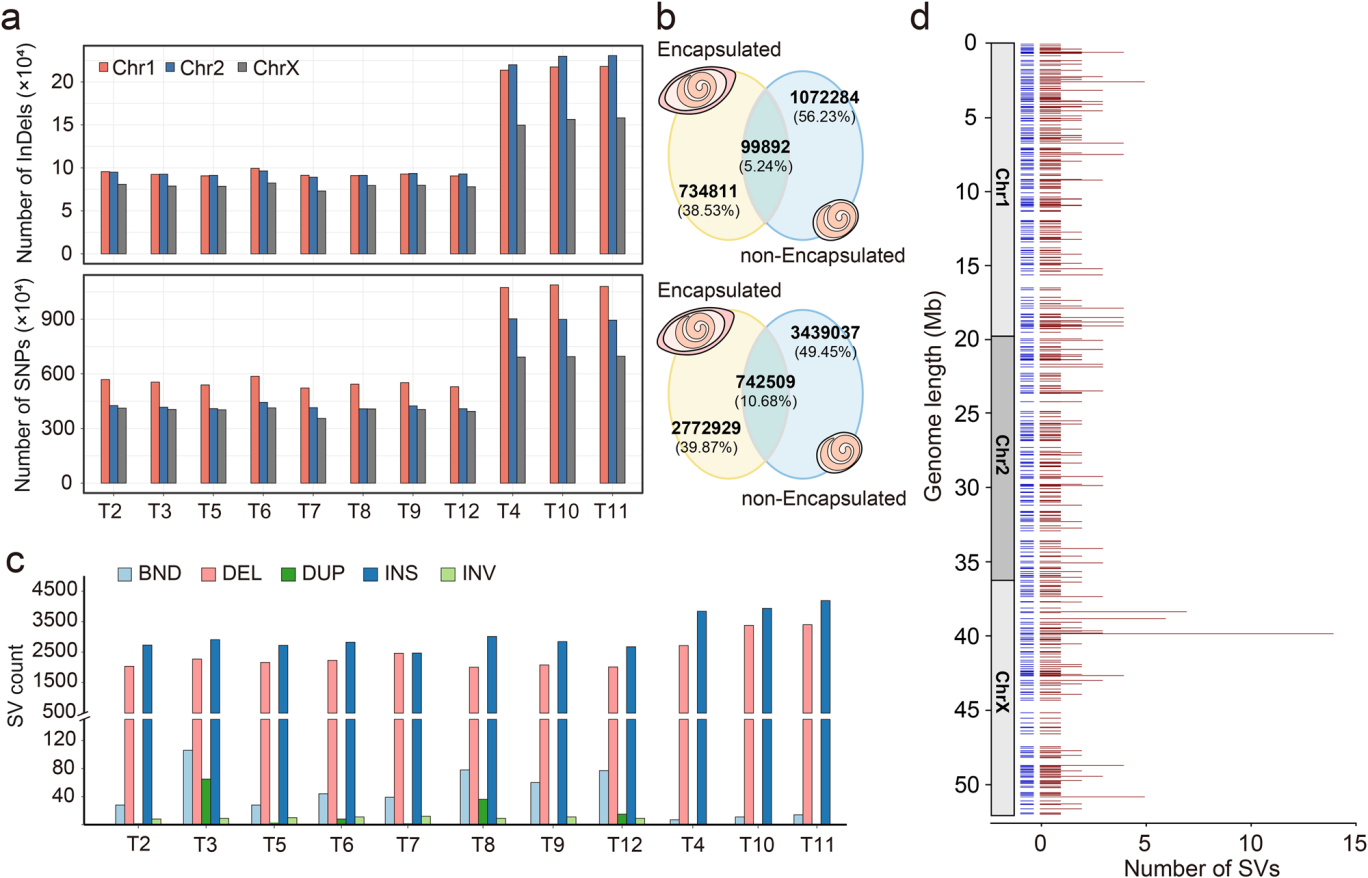
Genomic variation landscape of *Trichinella* species. **(a)** Counts of single nucleotide polymorphisms (SNPs) and InDels identified in each species relative to *T. spiralis*. (**b)** Venn diagram of shared and lineage‐specific SNPs/InDels between encapsulated and nonencapsulated clades. (**c)** Distribution of five structural variation (SV) types detected in each species compared with *T. spiralis*. (**d)** Chromosomal distribution of SV‐affected genes in the nonencapsulated clade. The blue tracks above the chromosomes indicate gene positions, with red peaks representing SV counts per gene.

Identifying the SVs in the genome of *Trichinella* is helpful for understanding and revealing the mechanism underlying the formation of the phenotype of the members of this genus. To construct a high‐quality variation catalog, we compared the T1 genome as a reference genome with those of other species as a reference for identifying genome‐wide variations. A total of 6.95 million nonredundant genetic variations (single nucleotide polymorphisms, SNPs) and 1.91 million small insertions and deletions (InDels) were identified. Chromosomes 1 and 2 had the highest densities of SNPs and InDels, respectively (Figure 5a, Table S14). We identified 159,830 high‐impact variations among 13,024 genes, corresponding to 42,171 SNPS and 117,659 InDels. In addition, we investigated the variations in efficiency within both encapsulated and nonencapsulated lineages. In addition to the SNPs/InDels shared by the two groups, 56.2% of the SNPs and 49.45% of the InDels originated from nonencapsulated species (Figure 5b). These results revealed extensive genomic variations in encapsulated and nonencapsulated species.

Comparative genomic analysis with the T1 reference genome revealed 21,319 nonredundant SVs, comprising 9333 deletions, 11,381 insertions, 534 breakends, 48 inversions, and 23 duplications (Figure 5c). Notably, nonencapsulated lineages presented the highest SV burden (Table S15). Annotation analysis revealed that 7764 high‐impact variants within these SVs affected 3194 protein‐coding genes. Specifically, in nonencapsulated lineages, we identified 1204 high‐impact SVs affecting 525 genes. The affected genes were primarily located on chromosome 1, while chromosome X exhibited a higher mutation density per gene (Figure 5d). Functional enrichment analysis revealed that these SV‐associated genes were involved in critical biological processes, including microtubule binding (GO:0008017), apical plasma membrane (GO:0016324), methyltransferase activity (GO:0008168), and methylated histone binding (GO:0035064) (Figure S7). In addition, these genes were involved in the cytoskeleton in muscle cells, nicotinate and nicotinamide metabolism, and the notch signaling pathway.

### 2.5 Transcriptional Expression Profile Reveals Cyst‐Linked Gene Families

**FIGURE 6:**
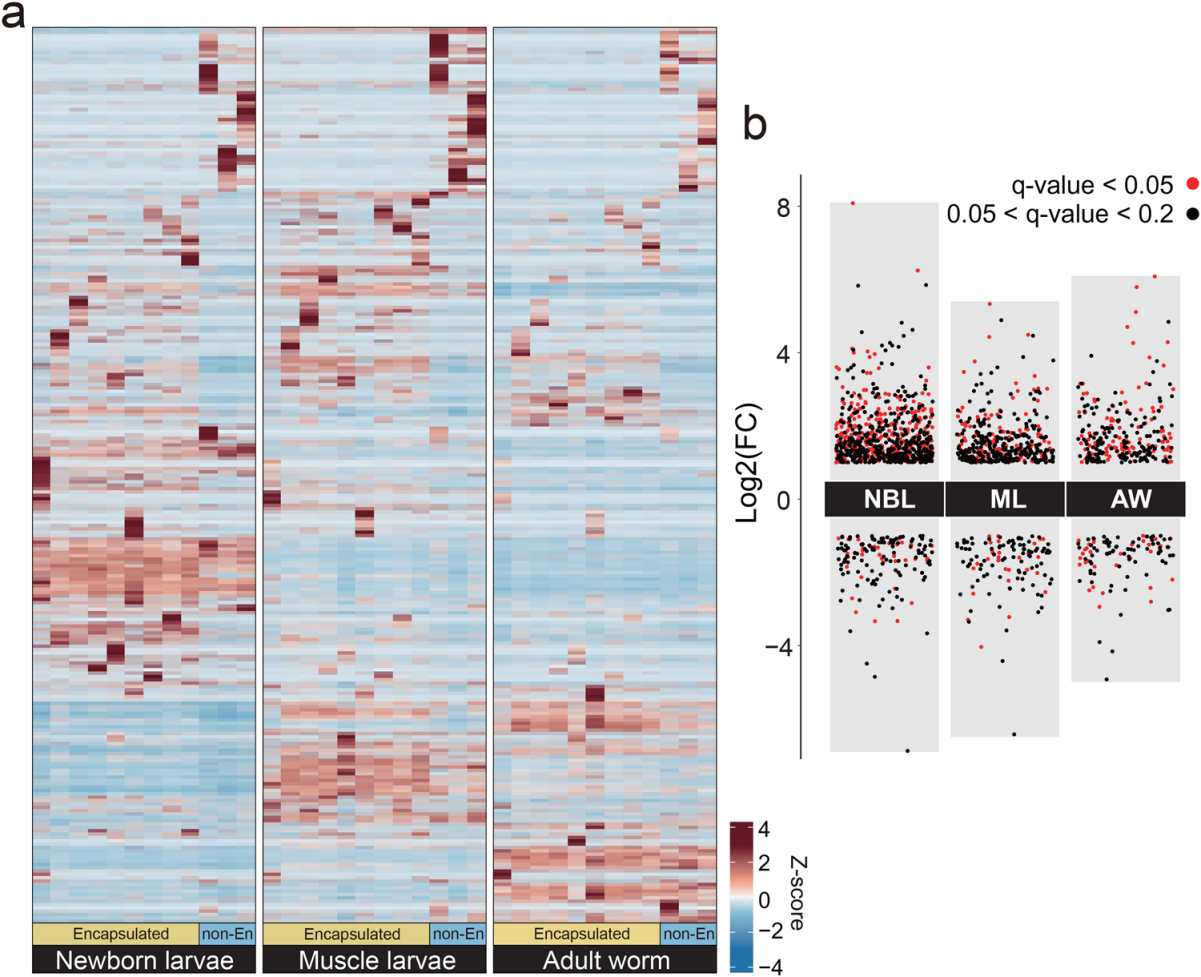
Stage‐specific transcriptomic profiling of *Trichinella* species. **(a)** Heatmap clustering of gene expression across three key developmental stages. Genes with similar expression patterns form stage‐specific clusters, revealing distinct transcriptional profiles between encapsulated (red) and nonencapsulated (blue) clades. (**b)** Differentially expressed genes between lineages across developmental stages. The upper scatter points represent genes that are upregulated in encapsulated lineages, whereas the lower points represent genes that are upregulated in nonencapsulated lineage‐specific lineages.

To investigate transcriptional divergence across species, we developed an analytical pipeline integrating gene family expression profiles from 12 species at three developmental stages (NBL, ML, and AW; Figure S8). Comparative analysis revealed conserved transcriptional patterns during development (Figure S9), with stage‐specific expression clusters reflecting parasite life cycle adaptations (Figure 6a). We identified three stage‐associated gene family clusters: NBL (1331 families), ML (759 families), and AW (947 families) (Figure S10; Table S16). The NBL cluster was enriched in transmembrane signaling and cellular dynamics, including G protein‐coupled receptor‐mediated signal transduction, calcium/potassium ion transport regulation, and cilia/axon development. The ML cluster was functionally linked to ribosomal biogenesis, mitochondrial homeostasis, and lysosomal activity. The AW cluster was enriched for host–parasite interaction mechanisms and reproductive maturation processes, notably serine/threonine phosphatase activity, protease‐adhesion systems, and structural biosynthesis pathways (Figures S11 and S12).

Comparative transcriptomic profiling revealed substantial divergence between the encapsulated and nonencapsulated lineages at the same developmental stage (Figures 6a and S13). These findings prompted our hypothesis that differentially expressed gene families across lineages might underlie pathways associated with cyst formation (Figure 6b, Table S17). In the ML stage, nonencapsulated species presented upregulated expression of genes encoding proteins with metalloendopeptidase activity (GO:0004222) and cortical cytoskeleton organization functions (GO:0030864) (Figure S14), indicating that active larvae promoted intratissue migration and invasion through extracellular matrix degradation. This phenomenon contrasted sharply with that of encapsulated lineages, which prioritized structural persistence mechanisms, such as the anaphase‐promoting complex (GO:0005680) and ribosome biogenesis (GO:0042254).

### 2.6 Host–Parasite Protein Interaction Network Implicates Multiple Pairs of Potential Interactors

**FIGURE 7:**
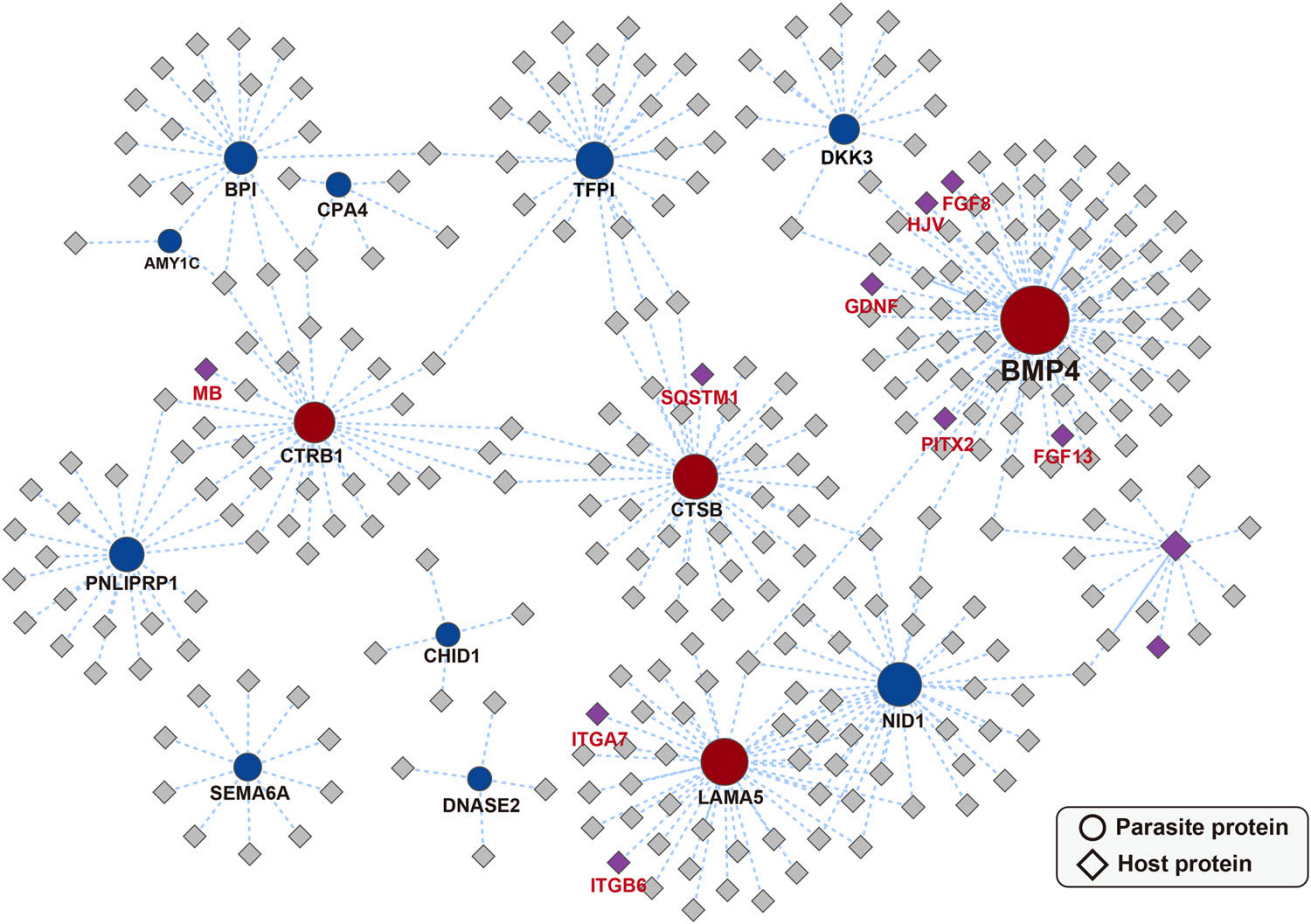
Host–parasite interaction network. Protein–protein interaction network between *Trichinella* secreted proteins and the human host. Red circles represent parasite‐secreted proteins interacting with human skeletal muscle‐enriched proteins; blue circles denote parasite‐secreted proteins interacting with other human proteins. Purple diamonds indicate human skeletal muscle‐enriched proteins, and gray nodes represent other human proteins.

To elucidate the protein–protein interactions between *Trichinella* and its host during NC formation, we constructed a host–parasite interaction network. A defined larval secretome and a host surface‐exposed proteome were used as inputs. Our analysis revealed potential interactions between proteins secreted by 18 parasites and 592 human host proteins (Figure 7, Table S18). Notably, four key parasite‐secreted proteins (BMP4, CTSB, LAMA5, and CTRB1) significantly interacted with nine host proteins involved in muscle‐related processes (FGF8, GDNF, HJV, PITX2, FGF13, ITGA7, ITGB6, MB, and SQSTM1), resulting in the formation of nine high‐confidence interaction pairs. Among these, BMP4 stood out for engaging in five distinct interactions. BMP4 is a pivotal growth factor known to regulate embryonic development, skeletogenesis, and tissue repair. Amino acid sequence alignment revealed that the TGF‐β‐like C‐terminal domain of BMP4 is completely conserved in 12 species of *Trichinella*, with only minor variations present in the TGF‐β propeptide region (Figure S15). We next performed pairwise structure alignment between the *T. spiralis* and human BMP4 proteins, which revealed a high degree of three‐dimensional similarity (TM score = 0.82; Figure S16). This structural conservation suggests that parasite‐derived BMP4 may functionally mimic its host counterpart to modulate host signaling pathways. Interestingly, we identified an 80–83 bp intronic insertion (T1‐Chr1:3165686‐3165686) in the *BMP4* gene which is present in all non‐encapsulating species but absent in encapsulating species (Figure S16, Table S19).

### 2.7 Cross‐Species Screening for Drug Targets

To rationally leverage this cross‐species genomic resource, we sought to identify potential drug targets conserved across *Trichinella* species. A total of 4077 single‐copy orthologous gene sets from 12 species were used for the systematic screening. In the primary screening, the union of ChEMBL database druggability genes and orthologs of lethal genes from WormBase yielded 774 unique genes (603 from ChEMBL and 549 from WormBase, with 378 genes overlapping between databases). These genes were advanced to a second filtering step, wherein homologs present in common host organisms (human, pig, and mouse) were excluded. This process resulted in a refined set of 28 genes (Table S20). Among these, 17 had a *S*_target_ score (as defined in the Methods section) greater than 4 (Table 3). Only *dpy‐31*, *rpb‐5*, and *mak‐1* were consistently identified in both databases. Of these, only *dpy‐31* had a high S_target_ score, thereby surpassing our most stringent selection criteria. The *dpy‐31* gene plays an essential role in *C. elegans* embryogenesis, and its homolog in parasitic nematodes has been implicated in their survival [15]. Consequently, *dpy‐31* was selected as the most promising candidate for further functional investigation.

**TABLE 3:** The identified candidates for drug targets in the *Trichinella*.

Gene	WBGene	chemblID	Uniprot	Gene Name	*S*_target_
unc‐15	00006754		P10567	Paramyosin	11.58525
dpy‐31	00006592	3739250	P98060	Zinc metalloproteinase dpy‐31	8.971953
inx‐1	00002123		Q17394	Innexin	6.025529
let‐522	00010870		Q21516	Uncharacterized protein	5.37567
F53E2.2	00302980		Q07750	Actin‐depolymerizing factor 1, isoforms a/b	5.110764
inx‐1	00002123		Q17394	Innexin	5.065584
tct‐1	00009122		Q93573	Translationally‐controlled tumor protein homolog	5.053086
sdhd‐1	00009353		O62215	Putative succinate dehydrogenase [ubiquinone] cytochrome b small subunit, mitochondrial	4.856029
inx‐1	00002123		Q17394	Innexin	4.81791
map3k10		6066128	Q0277	Mitogen‐activated protein kinase kinase kinase 10	4.767406
oig‐3	00003861		Q9NAF4	Ig‐like domain‐containing protein	4.587532
mrpl‐51	00011740		Q22438	Large ribosomal subunit protein mL51	4.583937
F52D10.2	00009930		Q20652	PID domain‐containing protein	4.427589
ost‐1	00003893		P34714	SPARC	4.311114
W07E11.1a	00012326		G5EF05	glutamate synthase (NADH)	4.204927
ndub‐10	00010326		Q93831	NADH dehydrogenase [ubiquinone] 1 beta subcomplex subunit 10	4.166713
mdt‐10	00007014		P45966	Mediator of RNA polymerase II transcription subunit 10	4.134528

**FIGURE 8:**
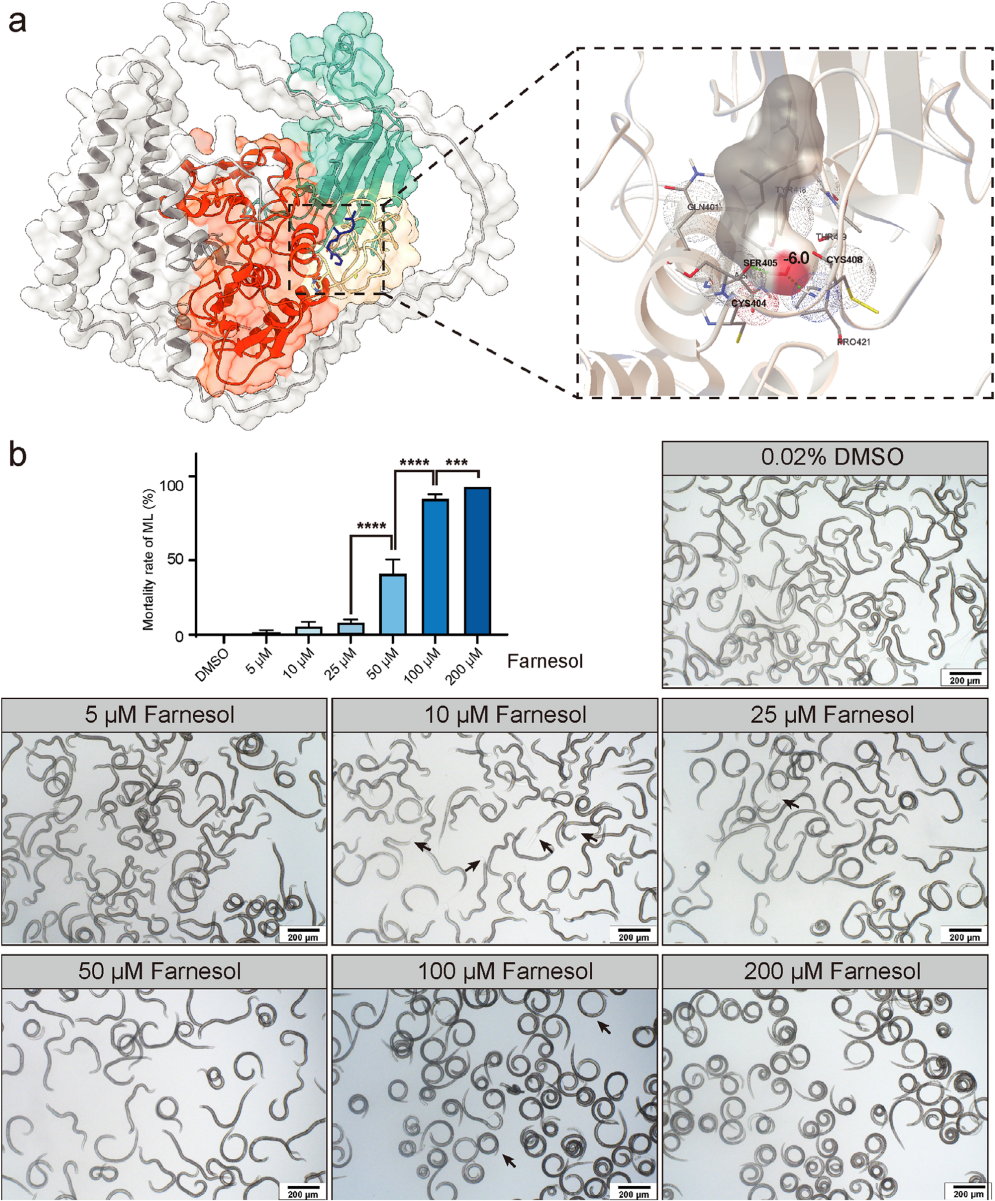
In vitro anthelmintic activity of farnesol against muscle larvae (ML) of *T. spiralis*. **(a)** Molecular docking model depicting the predicted binding pose of farnesol within the *T. spiralis* DPY‐31 protein. **(b)** Dose‐response effects of farnesol treatment on larval viability. Larval molting was observed in the control and low‐concentration groups (5–10 µM). Intermediate concentrations (25–50 µM) resulted in marked suppression of molting, while higher concentrations (≥100 µM) caused larval death, characterized by a distinctive C‐shaped or O‐shaped body curvature.

Although several known metalloprotease inhibitors have been reported to inhibit DPY‐31 effectively [16], their broad activity against host metalloproteases raises concerns about potential host toxicity. We note that farnesol, a natural sesquiterpenoid alcohol and an FDA‐approved food additive, is recognized for its safety profile [17]. Farnesol acts as a juvenile hormone analog and has been shown to inhibit nematode molting [18], a biological process linked to the function of DPY‐31. Molecular docking simulations revealed a binding affinity of approximately −6.0 kcal/mol between the *Trichinella* DPY‐31 protein and farnesol, suggesting a potential interaction (Figure 8a). We subsequently evaluated the in vitro anthelmintic activity of farnesol against ML. In the control groups, the larvae exhibited robust, random motility with negligible mortality after 24 h of exposure. In contrast, farnesol treatment resulted in dose‐dependent effects, with significant suppression of molting and reduced motility observed at concentrations above 25 µM (Figure 8b). At 100 and 200 µM, the mortality rates reached 95% and 100%, respectively.

## 3 Discussion

Owing to its obligate parasitic lifestyle and minute physical size, genomic studies of *Trichinella* have long been constrained by challenges in sampling and sequencing. To overcome these limitations, we established highly inbred lines through successive laboratory passages, effectively reducing individual heterozygosity. This strategy enabled the successful assembly of chromosome‐scale genomes for the majority of species within the genus.

The divergence between encapsulated and nonencapsulated lineages is estimated to have occurred approximately 18 million years ago (Mya) during the Miocene epoch. This timeframe aligns with recent recalibrations of nematode evolution and suggests that the diversification of *Trichinella* may have been influenced by global climatic shifts and the subsequent radiation of mammalian and avian hosts [10]. A notable radiation event between 2 and 5 Mya, which coincided with the Great American Biotic Interchange [19], supported the hypothesis that host migration events, coupled with geographical isolation and adaptive host switching, were key drivers of parasite speciation [7, 14]. During this period, large‐scale migrations of carnivorous mammals and birds likely facilitated ancestral host‐switching events [20], with subsequent host–parasite coevolutionary pressures propelling the speciation process [21].

Comparative genomic analyses revealed that compared with encapsulated lineages, nonencapsulated lineages possess a greater abundance of structural variants and gene families. these patterns are consistent with differential evolutionary constraints associated with host range and infection strategy. Nonencapsulated species infect a broader spectrum of hosts, including birds and reptiles [20, 22], which may permit greater genomic plasticity. In contrast, encapsulated lineages exhibit extensive gene family contraction, consistent with genomic simplification associated with long‐term persistence in mammalian muscle tissue. Encapsulation can be interpreted as an adaptive response to host immune pressure associated with long‐term tissue residence. Comparative analyses revealed that encapsulated lineages show strong signatures of positive selection in pathways related to membrane trafficking, ubiquitination, and Notch signaling, which are closely linked to host cell remodeling and maintenance of the NC environment [5, 23]. A compelling example is the E3 ubiquitin ligase RNF13 in *Trichinella*, which has been demonstrated to induce host myocyte differentiation via an autophagy‐dependent pathway [5], providing direct mechanistic evidence linking ubiquitination to the encapsulation process. In contrast, nonencapsulated lineages exhibit selection on metabolic pathways, consistent with alternative strategies for tissue invasion and persistence. These patterns suggest divergent evolutionary solutions to host‐associated selective pressures, although the precise immunological drivers remain to be elucidated.

Complementing these genomic patterns, a cross‐species transcriptomic atlas revealed conserved, stage‐specific expression clusters aligned with parasitic development. NBL‐stage genes, including G‐protein coupled receptors, are involved in larval migration and immune evasion during early infection [24]. ML‐stage gene clusters are enriched in functions related to ribosome biogenesis and lysosomal activity, reflecting the metabolic demands of cyst formation. Furthermore, AW‐stage genes, such as cysteine cathepsins, serine proteases, and metalloproteases, mediate reproduction and tissue penetration in the intestinal niche [25, 26, 27]. More importantly, we observed significant transcriptional divergence between encapsulated and nonencapsulated lineages at homologous developmental stages. For example, genes involved in metalloendopeptidase activity and cortical cytoskeleton organization were upregulated in the ML stage of nonencapsulated species, potentially enhancing their tissue migratory and invasive capabilities. In contrast, genes associated with structural maintenance and immunomodulation, such as ribosome biogenesis and ubiquitin‐dependent processes, were predominantly upregulated in encapsulated lineages.

A prominent evolutionary mechanism in host–pathogen dynamics is molecular mimicry, whereby pathogens exploit homologs of host immunomodulatory molecules to hijack native regulatory pathways and reshape the immune microenvironment to their advantage [28, 29]. To explore this in *Trichinella*, we constructed a predicted protein‐protein interaction network between the parasite and its host in accordance with the methods of Hu et al. [8]. Notably, such in silico approaches have inherent limitations, and the resulting network must be cautiously interpreted. Therefore, we applied stringent filters to focus on a high‐confidence subset of interactions. Our final candidate set consisted of secretory proteins that were highly expressed during the ML or NBL stages in all the encapsulated species but expressed at low levels in the nonencapsulated species. Furthermore, we restricted the host interactome to proteins highly expressed in human skeletal muscle rather than the entire human proteome. This refined methodology allowed us to narrowly focus on interactions potentially occurring during the critical transition from NBL to ML. While this conservative approach undoubtedly overlooks many genuine host–parasite interactions, it provides a robust, evidence‐based foundation for prioritizing key mediators of capsule‐associated molecular mimicry.

Among these candidates, the parasite BMP4 homolog emerged as a protein of interest due to its lineage‐biased expression, association with SV, and strong structural similarity to host BMP4. BMP and FGF signaling play crucial roles in early tissue patterning and differentiation [30]. BMP4 positively regulates FGF8, whose overexpression in turn promotes FGFR4 expression and muscle differentiation [31, 32]. Furthermore, FGF13 acts as a negative regulator of skeletal muscle development through the downregulation of Spry1 [33]. While these features raise the possibility that parasite‐derived BMP4 may interact with host developmental signaling pathways, this observation should be interpreted as hypothesis‐generating rather than evidence of functional molecular mimicry. Notably, although an intronic structural variant distinguishes encapsulated from nonencapsulated lineages, its regulatory impact on BMP4 expression remains untested and warrants future investigation.

The multiomics dataset, which integrates cross‐species and cross‐developmental information, provided an unprecedented resource for in silico drug target screening. Notably, our stringent filtering pipeline yielded only a single high‐confidence candidate drug target gene across the genus. DPY‐31, also known as NAS‐35, is a well‐documented essential protein in nematodes and is classified as a nematode astacin zinc metalloprotease [34]. It plays a critical role in cuticle synthesis for both free‐living and parasitic nematodes [15]. Mutations in *dpy‐31* are lethal in *C. elegans*, causing severe cuticle defects, and this gene has been previously proposed as a suitable drug target for several gastrointestinal nematodes [16]. While compound inhibitors targeting DPY‐31 have been identified, their practical application remains challenging [16]. We therefore turned our attention to a potential inhibitor consistent with the functional range of DPY‐31. Farnesol, a reported juvenile hormone analog, was previously shown to inhibit *Trichinella* molting and the development of male copulatory structures [18]. In vitro assays confirmed that farnesol is a potent molting inhibitor and larvicide. Subsequent molecular docking simulations suggested a potential interaction between farnesol and DPY‐31. We acknowledge, however, that the specific mechanism of action may be more complex, and we cannot definitively conclude whether the observed effects are mediated directly through DPY‐31 inhibition. Nevertheless, the low cost of farnesol and its established safety profile, render it an attractive candidate for further development as an anthelmintic agent.

The functional validation of candidate genes in *Trichinella* remains challenging because of the current lack of robust, stable in vitro culture systems or genetic tools. The inapplicability of current gene‐knockout techniques underscores this critical limitation. Future efforts must prioritize breakthroughs in in vitro culture and the development of a CRISPR‐based gene‐editing platform to accelerate functional genomics. Furthermore, expanding population genomic analyses to include geographically diverse isolates could reveal finer‐scale adaptations and evolutionary mechanisms based on host specificity [35, 36, 37]. In summary, this work not only advances our understanding of *Trichinella* evolution and encapsulation biology but also provides a foundational toolbox for future functional studies and intervention strategies. These resources advance functional genomics and target discovery, exemplifying how whole‐genome approaches can elucidate complex host–parasite interactions and antagonistic coevolution in parasitic helminths.

## 4 Methods and Materials

### 4.1 Parasite Collection and Extraction of Genomic DNA and RNA

A total of 12 recognized *Trichinella* species or genotypes used in this study were maintained and passaged in the laboratory. The samples contained the following strains: *T. spiralis* (ISS534), *T. nativa* (ISS70), *T. britovi* (ISS100), *T. pseudospiralis* (ISS141), *T. murrelli* (ISS35), *Trichinella* T6 (ISS34), *T. nelsoni* (ISS37), *Trichinella* T8 (ISS124), *Trichinella* T9 (ISS408), *T. papuae* (ISS1980), *T. zimbabwensis* (ISS1029), and *T. patagoniensis* (ISS1826). All the strains were maintained via serial passages in female ICR mice. ML and AW were isolated using established protocols [13]. Male AWs were manually sorted under a stereomicroscope by two trained technicians, with each sample aliquot containing 1000 individuals. Upon collection, all the samples were immediately flash‐frozen in liquid nitrogen and stored at −80°C until nucleic acid extraction.

Genomic DNA was isolated from all the samples using an SDS‐based lysis buffer protocol, followed by phenol‐chloroform purification and ethanol precipitation. Purified DNA was eluted in sterile buffer and quantified via Nanodrop (Thermo Fisher Scientific, USA) spectrophotometry. RNA extraction was performed using TIANGEN TRNzol Universal Reagent (TIANGEN Biotech, Beijing, China), which incorporates DNase I treatment to eliminate genomic DNA contamination. All the samples were stored at ‐80°C until library preparation.

### 4.2 Whole‐Genome Sequencing

Whole‐genome sequencing was performed using complementary short‐read and long‐read platforms. DNA libraries for next‐generation sequencing were prepared with the NEBNext Ultra II DNA Library Prep Kit (New England Biolabs, Ipswich, MA, USA), incorporating enzymatic fragmentation (350‐bp target inserts), end‐repair/dA‐tailing, and dual‐index adapter ligation. Libraries were subjected to quality control via an Agilent 2100 Bioanalyzer (Agilent, USA), which confirmed the 450‐550‐bp fragment distributions, and sequenced on the Illumina NovaSeq 6000 platform (Illumina, USA) using a 150‐bp paired‐end (PE150) strategy. Raw sequencing data were subjected to rigorous quality control using Fastp v0.23.4 with default parameters [38]. For long‐read scaffolding, size‐selected SMRTbell libraries (with 10‐kb and 20‐kb inserts) were constructed using the SMRTbell Express Template Prep Kit 2.0 (Pacific Biosciences, Menlo Park, CA, USA). The libraries were loaded onto SMRT Cell 8 M chips and sequenced on the PacBio Sequel IIe system to generate continuous long reads (CLR). To resolve the chromosomal architecture, Hi‐C libraries were prepared following the optimized protocol of Belton et al. [39]. The process involved library construction and PCR amplification (12–14 cycles) prior to NovaSeq 6000 PE150 sequencing, ensuring multiplatform genomic coverage for comprehensive assembly.

### 4.3 Genome Assembly and Quality Assessment

Before assembly, k‐mer frequencies were determined using Jellyfish v2.3.1 (with a k‐mer size of 17) [40]. On the basis of short‐read sequencing data, GenomeScope 2.0 was used to estimate the genome size, repeat ratio, and heterozygosity rate per species [41]. For each species, long‐read data were independently de novo assembled using NextDenovo v2.5.2 [42] and Canu v2.2 [43], and the key parameters for NextDenovo included the following: nextgraph_options = ‐a 1 ‐q 10. The resulting assemblies were processed with Purge Haplotigs v1.1.3 [44] to remove heterozygous redundancies, followed by four rounds of polishing with NextPolish v1.4.1 [45]. Potential contaminant sequences were identified and removed via FCS‐GX v0.5.4 [46]. The two de novo assemblies were merged into a final assembly using QuickMerge v0.3 [47]. To construct chromosome‐scale scaffolds for all 12 species, the Hi‐C data were analyzed. Scaffold‐level assemblies were processed using Juicebox v1.20.00 [48] and the 3D‐DNA v201008 pipeline [49]. Genome completeness was ultimately assessed with BUSCO v5.7.1 [50], utilizing both the *eukaryota_odb10* and *nematoda_odb10* datasets. The publicly available reference genome (NCBI accession numbers PRJNA12603 and PRJNA257433) [12, 14] and the newly assembled genomic indicators were determined using the same script and software, respectively.

### 4.4 Identification of Repeats

To identify whole‐genome repeats, a combined strategy based on homology alignment and a de novo search was employed. For homology‐based detection, known repeat regions were annotated using RepeatMasker v4.1.6 (http://www.repeatmasker.org) with the RepBase database [51]. De novo repeat libraries were constructed using LTR_FINDER v1.3 [52], RepeatScout v1.0.6 [53], and RepeatModeler v2.0.5 [54]. All the predicted repeat sequences with lengths >100 bp and ≤5% ambiguous bases (‘N’) were used to construct the raw transposable element (TE) library. A custom nonredundant library was generated by merging RepBase entries with our novel TEs and supplied to RepeatMasker for DNA‐level repeat identification.

### 4.5 Gene Prediction and Annotation

Structural annotation of the genomes was performed using an integrative approach that combined ab initio prediction, homology‐based prediction, and RNA‐Seq evidence. For structural gene prediction, repeat soft‐masked genomes were analyzed using both the BRAKER v3.0.8 [55] and MAKER v3.01.04 [56] pipelines. All the *Trichinella* protein sequences in the UniProtKB database [57] were provided as evidence to the pipelines. For cross‐species homology prediction, Liftoff v1.6.3 [58], GeMoMa v1.9 [59], and Exonerate v2.4.0 [60] were employed, with each species annotated using its closest available reference genome. Transcriptome assemblies were generated using Trinity v2.15.1 [61] and subsequently processed through PASA v2.5.3 [62] for model training. RNA‐Seq reads were aligned to the genome using HISAT2 v2.2.1 [63], with default parameters for identifying exon boundaries and splice sites. Transcriptome‐based gene structure predictions were performed using StringTie v3.0.0 [64]. Predictions from all the approaches were consolidated using EvidenceModeler v2.1.0 [65] to generate a nonredundant, high‐confidence reference gene set. The AGAT toolkit v1.4.1 (https://github.com/NBISweden/AGAT) was used to collect genomic structure data, including the number of genes and the exon and intron lengths. Finally. The completeness of the genome assemblies was assessed using BUSCO v5.7.1 [50] in protein mode.

The functional annotation of the predicted gene models was performed using a comprehensive bioinformatics pipeline. Protein homology searches were conducted using DIAMOND v2.1.9.163 [66] against the NCBI NR database (release 2024‐02‐07) with a stringent *E* value <1 × 10^−5^. Protein domains and motifs were identified using InterProScan v5.73‐104.0 [67], which queried multiple specialized databases, including Pfam, PRINTS, PROSITE, ProDom, SMART, and PANTHER [68]. Gene Ontology (GO) terms were subsequently assigned on the basis of the InterPro annotations. Metabolic pathway analysis was performed using KofamScan v1.3.0 [69] with an *E* value <1 × 10^−5^ to assign KEGG Orthology (KO) identifiers. Small noncoding RNAs (ncRNAs), including microRNAs (miRNAs), tRNAs, rRNAs and small nuclear RNAs (snRNAs), were identified using Infernal v1.1.5 [70] with default parameters against the Rfam database (release 15.0) (https://rfam.org/) [71]. Genomic features were visualized using TBtools to facilitate comparative analysis [72].

### 4.6 Synteny Analysis

Synteny among *Trichinella* genomes was investigated using GENESPACE v1.2.3 [73] with OrthoFinder v2.5.5 in ‘default’ mode [74].

### 4.7 Gene Family Analysis

Orthologous gene clusters across the assembled genomes were identified using OrthoFinder v2.5.5 [74] with default parameters. These clusters formed the basis for subsequent phylogenetic reconstruction: a maximum‐likelihood tree was generated using RAxML v1.2.2 [75] on the basis of conserved single‐copy orthologs from the OrthoFinder results. Divergence time estimation between species was performed via Bayesian analysis using MCMCtree (PAML package v4.10.7) [76]. To delineate the evolutionary dynamics of gene families, CAFE5 v1.1 [77] was employed to detect lineage‐specific expansion/contraction events at each speciation node, while positive selection pressure in foreground branches was assessed using branch‐site models in CodeML (PAML package v4.10.7) [76]. Genome‐wide evolutionary rates (d*N*/d*S* ratios) were calculated through the global mode (module = 0) of CodeML. Pangenome and core‐genome profiles were established through comparative analyses using custom R scripts, and functional enrichment of gene families was subsequently evaluated by clusterProfiler v4.12.6 [78] and visualized using ggplot2 v3.5.1 [79].

### 4.8 Detection of Genomic Structural Variations

Single nucleotide polymorphisms (SNPs) and small indels (<50 bp) were identified using the show‐snps utility from MUMmer4 v4.0.1 [80] with the parameters ‘‐ClrT’. These variants were functionally annotated using SnpEff v5.2f [81], whose effects on coding and regulatory regions were predicted. Whole‐genome alignments between the 11 *Trichinella* genomes and the T1 reference were performed using Minimap2 v2.28‐r1209 [82] under the asm20 preset (‐x asm20 ‐cs ‐r2k), allowing ≤20% sequence divergence. SVs were detected using Svim‐asm v1.0.3 [83], followed by consensus SV merging across species with SURVIVOR v1.0.7 [84] (merge distance: 100 bp; parameters: ‘100 1 1 50’). Finally, SV annotations, including potential gene disruptions, were generated through SnpEff v5.2f [81]. In addition, VCF files were processed and filtered using BCFtools v1.21 [85].

### 4.9 Transcriptome Analysis

A standardized RNA‐seq processing pipeline was implemented to generate cross‐species transcriptional profiles. Raw reads were aligned to their respective reference genomes using HISAT2 [63] with splice‐aware mapping parameters. Transcript abundance was quantified using StringTie [64], which employs normalized transcripts per million (TPM) values. Gene expression matrices across all 12 species were consolidated on the basis of orthologous gene families identified by OrthoFinder v2.5.5 [74] to construct a pangenus transcriptional atlas of *Trichinella*. Subsequent analyses were performed using an integrated R workflow: DESeq2 v1.38.3 [86] for differential expression analysis, edgeR v3.40.2 [87] for normalization, vegan v2.6‐10 [88] for principal component analysis (PCA) and multivariate statistics, and ggplot2 v3.5.1 [79] and ComplexHeatmap v2.14.0 [89] for hierarchical clustering visualization. Data transformation and format harmonization were facilitated via reshape2 v1.4.4 [90].

### 4.10 Construction of the Host–Parasite Protein Interaction Network

Based on transcriptomic analysis, we selected gene families that were upregulated in encapsulated species relative to non‐encapsulated species in both the NBL (133 families) and ML (332 families) stages (Table S17). According to these gene families, a representative set of 575 proteins from *T. spiralis* was selected, and 36 secreted proteins were identified from these proteins to constitute the parasite secretome. A set of 921 genes highly expressed in human skeletal muscle was obtained from the Human Protein Atlas (https://www.proteinatlas.org/) [91]. This protein set was subsequently mapped onto the human protein–protein interaction network from the STRING database [92], resulting in a host interaction proteome including muscle‐enriched proteins and their direct interacting proteins.

Homology between parasite proteins and host proteins was identified based on reciprocal best hits using BLAST analysis with an E‑value cutoff of 1e‑5. Host–parasite protein interactions were inferred by transferring the interaction partners of host proteins to their parasite‐encoded orthologs. Specifically, if a host protein X and a parasite protein X' were orthologs, the parasite protein X' was assumed to inherit the interaction network of host protein X. Only interactions with a STRING confidence score greater than 0.7 were retained. The resulting interaction network was visualized using Cytoscape 3.9.1.

### 4.11 Comparative Structural Analysis of BMP4

The 3D structural model for the BMP4 homolog of *T. spiralis* was retrieved from the SWISS‐MODEL repository (UniProt ID: D2DEC8), and the human BMP4 model was obtained similarly (UniProt ID: P12644). Pairwise structure alignment was performed using the jCE‐CP alignment method via the PDB server's online analysis tools (https://www.rcsb.org/alignment) [93].

### 4.12 In Silico Screening for Potential Drug Targets

Following the methodology outlined by Zhang et al. [29], we performed an in silico screening for potential drug targets within the single‐copy orthologous gene sets of *Trichinella*. Essential nematode genes were obtained from WormBase (WBPhenotype:0000062, release WS226). Host gene sets (human, pig, and mouse) were sourced from the NCBI Reference Sequence Database (GCF_000001405.40_GRCh38, GCF_000003025.6_Sscrofa11.1 and GCF_000001635.27_GRCm39). Three criteria were applied to compare host and parasite orthologs: query coverage (qcovs), subject coverage (scovs), and percentage identity (pident). 1) Lethal genes meeting (pident ≥ 50%) AND (qcovs ≥ 50% OR scovs ≥ 50%); 2) Druggability genes meeting (pident ≥ 50%) AND (qcovs ≥ 50% OR scovs ≥ 50%) with targets in the ChEMBL database; 3) Parasite specificity genes exhibiting low similarity to host orthologs, defined as (pident < 50%) OR (qcovs < 50% AND scovs < 50%).

A composite score (*S*_target_) was calculated to rank the identified candidates using the following formula:

(1) $$S_{\mathrm{target}}=\left(S_{l}+S_{d}\right)\times 2+S_{t}+S_{e}$$

(2) $$S_{t}=\frac{\log\left(T\right)}{\log\left(10\right)}$$

where *T* is the mean TPM value of the gene across the 12 species and *S*_l_ and *S*_d_ are the normalized alignment scores (S/L) for the “Lethal” and “Druggability” categories, respectively, with S representing the BLAST bit score and L representing the query sequence length. An enrichment score (*S*_e_ = 1) was assigned if the gene encoded a protease, kinase, phosphatase, transporter, or ion channel; otherwise, *S*_e_ was 0.

Finally, a three‐tiered screening strategy was implemented: 1) The “Druggability” and “Lethal” gene sets were merged to generate a broad initial candidate list. 2) To minimize potential host cross‐reactivity, the intersection between the merged set and the “Parasite‐specific” gene set was identified. 3) The final gene set was ranked by the *S*_target_ score. Candidates present in both the “Druggability” and “Lethal” datasets and possessing a high *S*_target_ score were considered to have passed the most stringent selection criteria.

### 4.13 Molecular Docking of DPY‐31 with Farnesol

The 3D structure of the *T. spiralis* DPY‐31 protein was predicted using AlphaFold3 [116]. The 3D structure of farnesol was retrieved from the PubChem database. File format conversions were performed using Open Babel v3.1.1 [94]. Molecular docking simulations were conducted using the AutoDock Vina engine [95], with 1000 independent runs performed. The conformation with the lowest binding energy was selected for analysis. Visualization of the protein–ligand complex was carried out using ChimeraX 1.10.1 [96].

### 4.14 In Vitro Assessment of Farnesol Toxicity Against ML

Under aseptic conditions, ∼7000 MLs were evenly divided into seven groups: a negative control group (0.02% DMSO) and six treatment groups (0.02% DMSO supplemented with 5, 10, 25, 50, 100, or 200 µM farnesol). ML in each group was incubated in 2 mL of DMEM (not contain serum) supplemented with 1% penicillin‐streptomycin at 37°C with 5% CO_2_. ML viability was assessed after 12 h. An ML was considered dead if it was immotile and exhibited a characteristic “C”‐ or “O”‐shaped body curvature. For quantification, two researchers, who were blinded to the experimental group assignments, randomly counted 100 larvae per group, and this process was repeated five times.

### 4.15 Statistics and Reproducibility

Statistical analyses throughout this study rigorously applied parametric assumptions, including verification of normality and homogeneity of variance. When these assumptions were not met, non‐parametric alternatives (Wilcoxon rank‐sum test) were employed. Functional enrichment analysis of target genes relative to the background was performed using a two‐tailed adjusted Fisher's exact test. For other comparative analyses, two‐tailed Student's *t*‐tests were used. Significance thresholds were defined as follows: ^*^ *p* < 0.05, ^**^ *p* < 0.01, and ^***^ *p* < 0.001.

## Funding

This study was supported by The National Key Research and Development Program of China (2023YFD1801000), National Natural Science Foundation of China (NSFC32373032 and 32230104) and Science and technology talents and platform plan of Yunnan province (Academician and Expert Workstation, 202305AF150167).

## Conflicts of Interest

The authors declare no conflicts of interest.

## Supporting information




**Supporting File 1**: advs75236‐sup‐0002‐TableS1.xlsx.

**Supporting File 2**: advs75236‐sup‐0001‐FigureS1.pdf.

## Data Availability

The data supporting the findings of this study have been deposited in public repositories. The raw sequencing data, including Illumina short‐reads, PacBio long‐reads, and Hi‐C data, have been submitted to the NCBI Sequence Read Archive (SRA) under the BioProject accession number PRJNA1336608. The assembled and annotated genomes are available under the same project. The transcriptomic data analyzed in this study were derived from the published work of Feng et al., which is accessible at GEO under accession number GSE140382.
