# Supplementary material for: The Trichinella Super‐Pangenome Reveals the Evolution of Encapsulation and Predicted Host–Parasite Protein Interactions
Source: Adv Sci (Weinh). 2026 Apr 10:e23161. Online ahead of print. doi: 10.1002/advs.202523161 (PMC13334664; doi:10.1002/advs.202523161)
Supplement: Supplementary file 2 — Supporting File 2: advs75236‐sup‐0001‐FigureS1.pdf. [file ADVS-9999-e23161-s001.pdf]

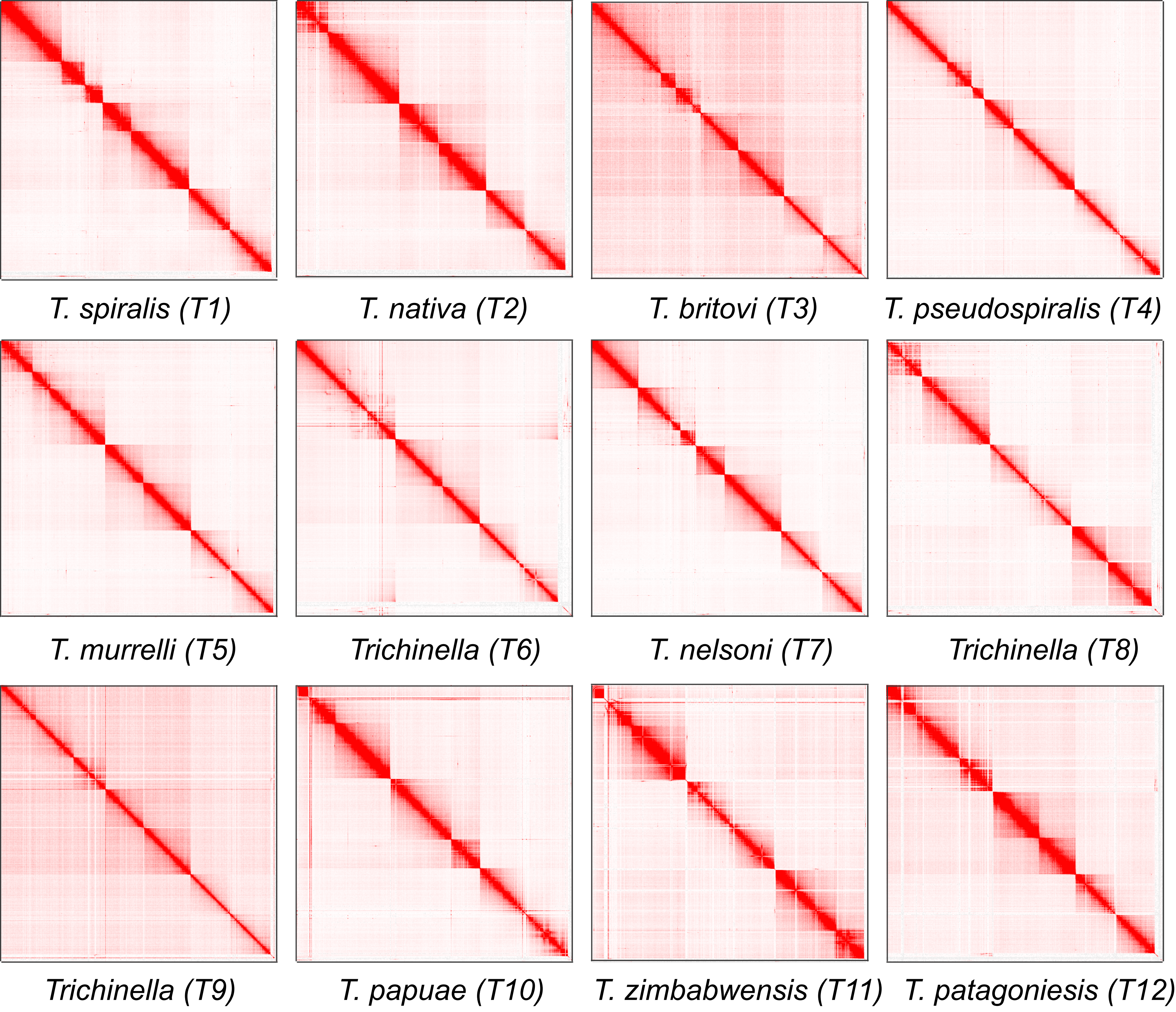

Fig. S1 Hi-C contact matrices for 12 Trichinella genome assemblies

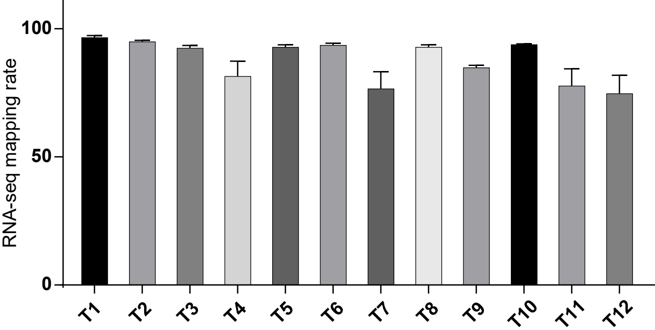

Fig. S2 RNA-seq mapping rate of 12 *Trichinella* genome assemblies

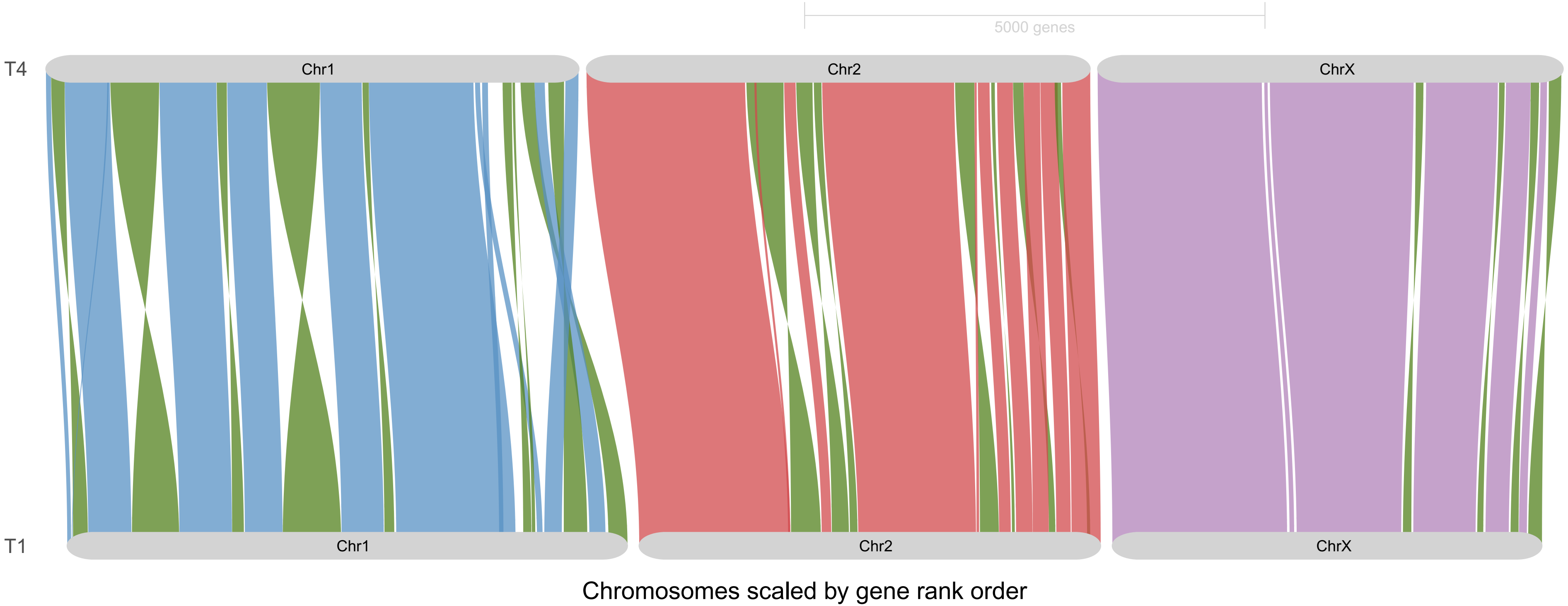

Fig. S3 Synteny analysis of *T. spiralis* and *T. pseudospiralis*

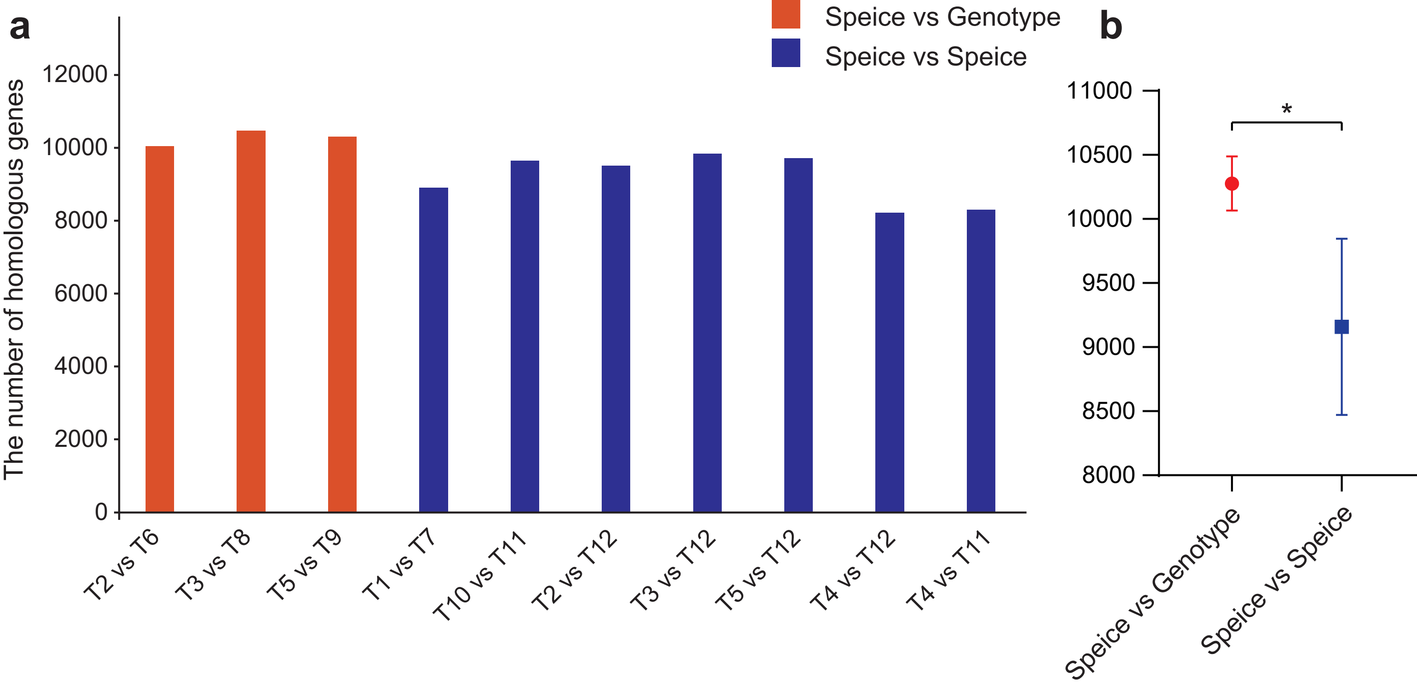

Fig. S4 Shared orthologous genes among Trichinella species. a, Number of orthologous genes shared between phylogenetically related species or genotypes. **b,** Comparative analysis of orthologous gene counts between species-genotype pairs and species-species pairs.

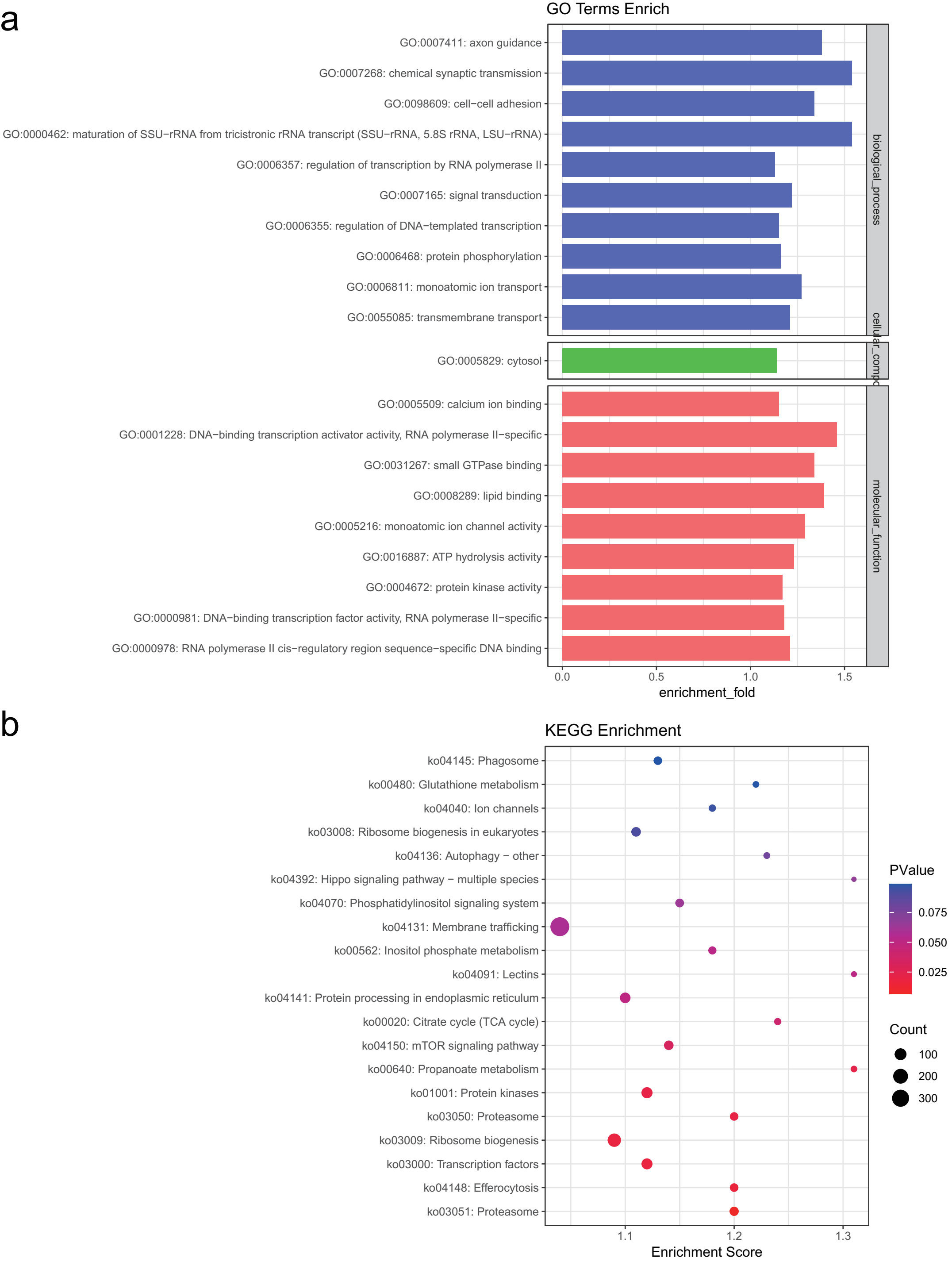

Fig. S5 Functional enrichment analysis of core gene families in Trichinella.a, Gene Ontology (GO) enrichment analysis of conserved core genes. **b,** KEGG pathway enrichment of core genes.

a

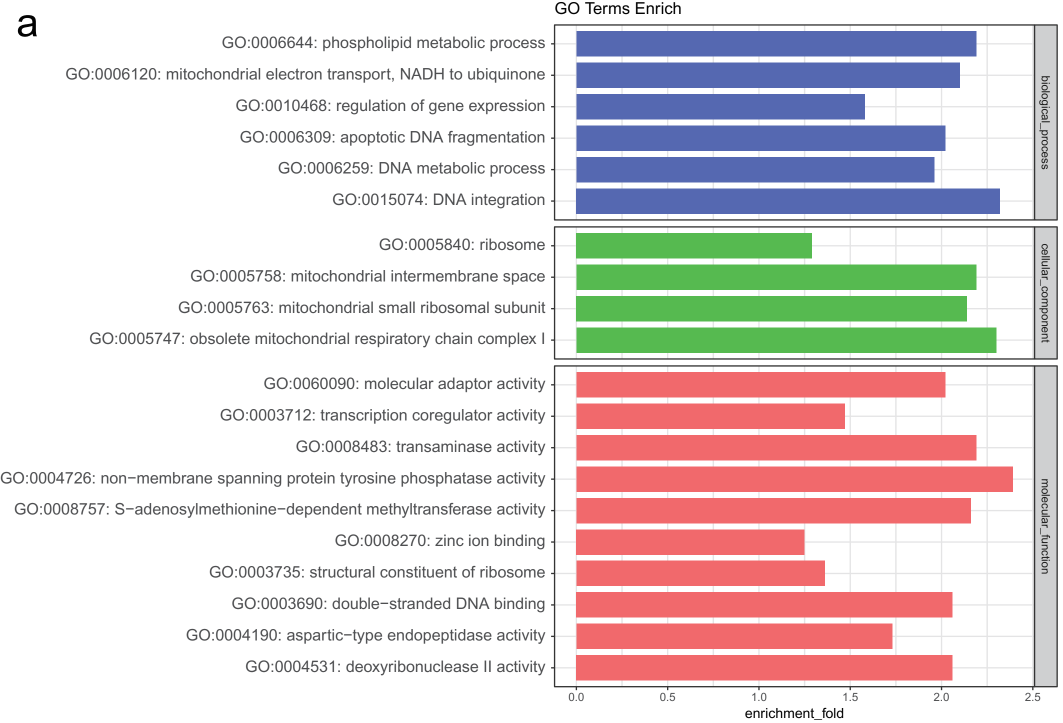

b

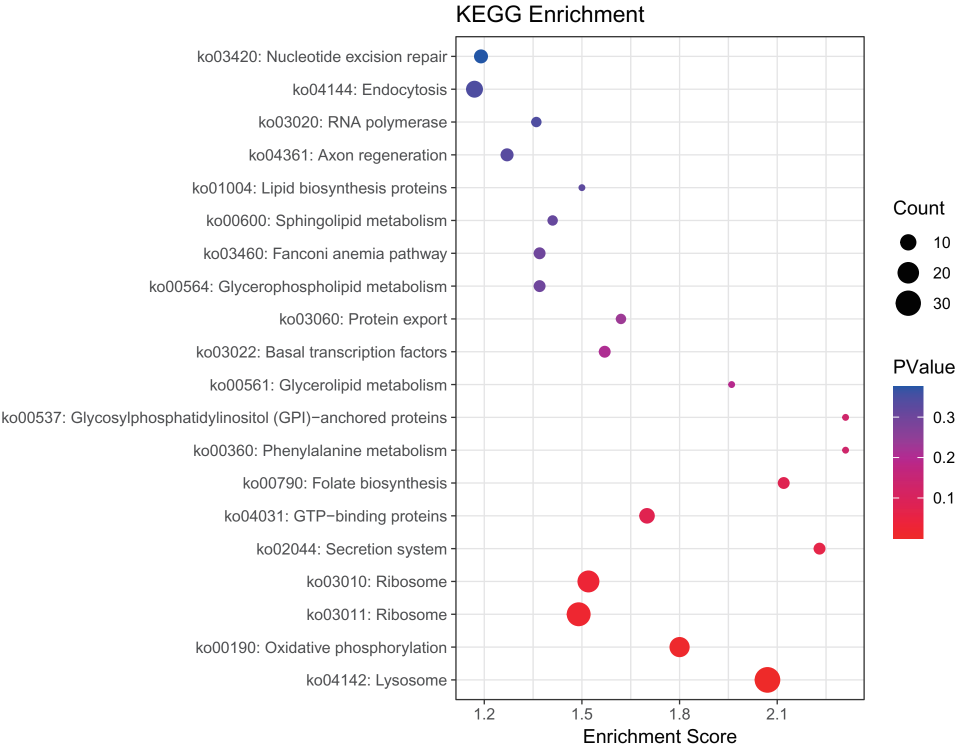

Fig. S6 Functional enrichment analysis of dispensable gene families in *Trichinella*. a, GO enrichment analysis of dispensable genes. **b,** KEGG pathway enrichment of dispensable genes.

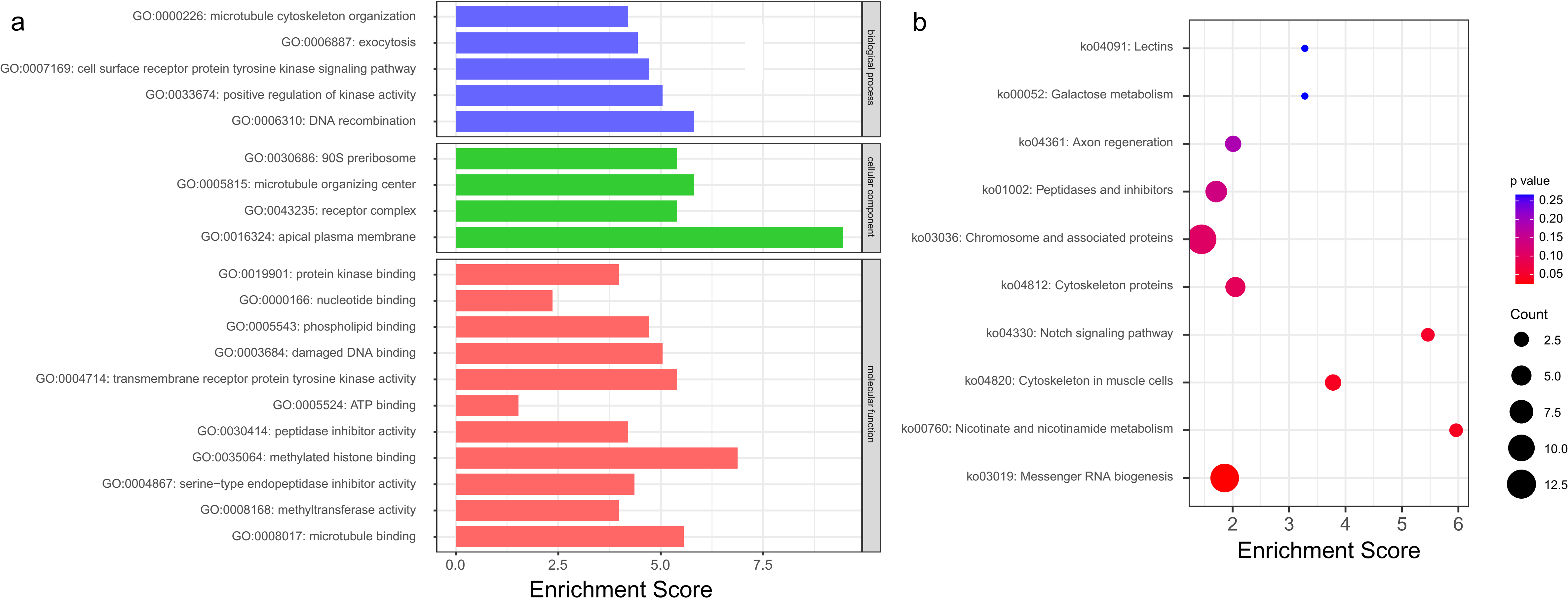

Fig. S7 Functional enrichment analysis of SV-associated genes. a, GO enrichment analysis of SV-associated genes. **b,** KEGG pathway enrichment of SV-associated genes.

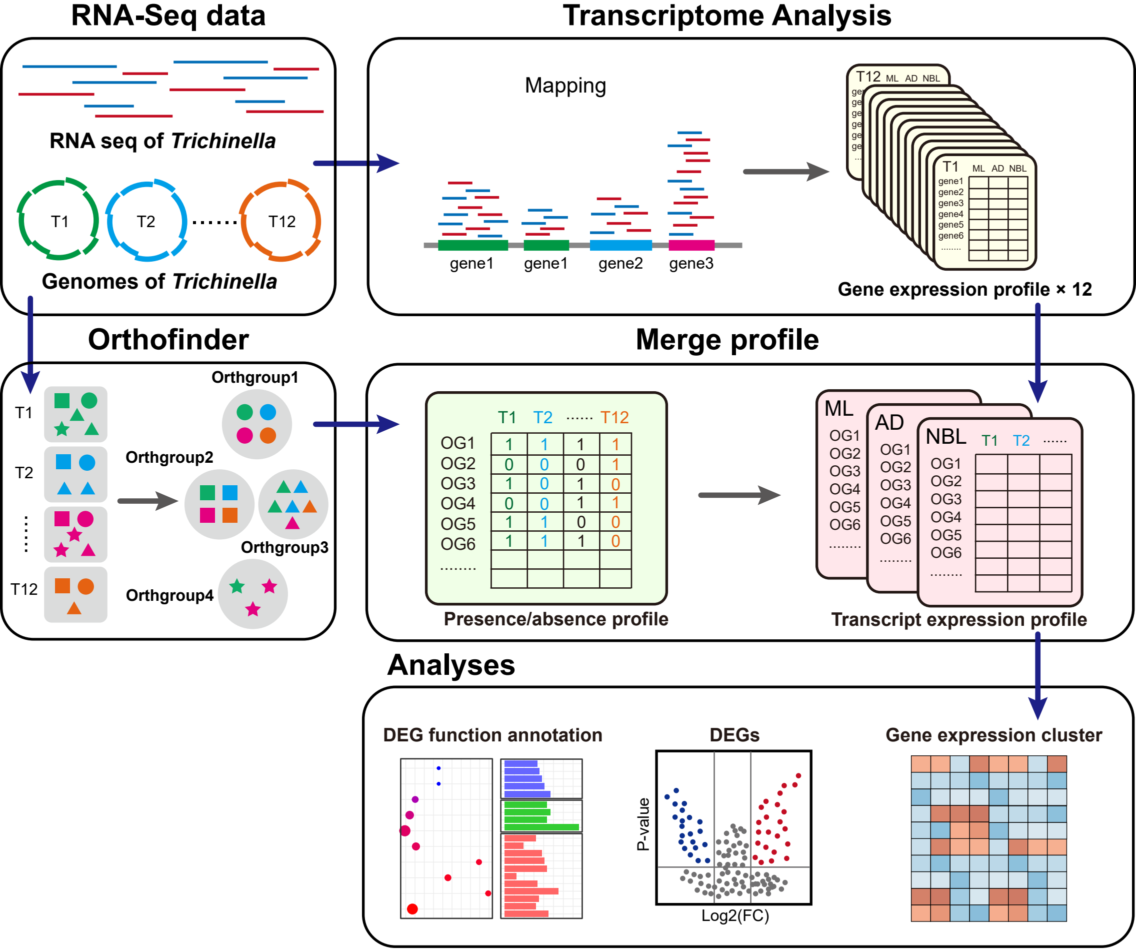

Fig. S8 The process pipeline for the combination and analysis of multi-species transcription.

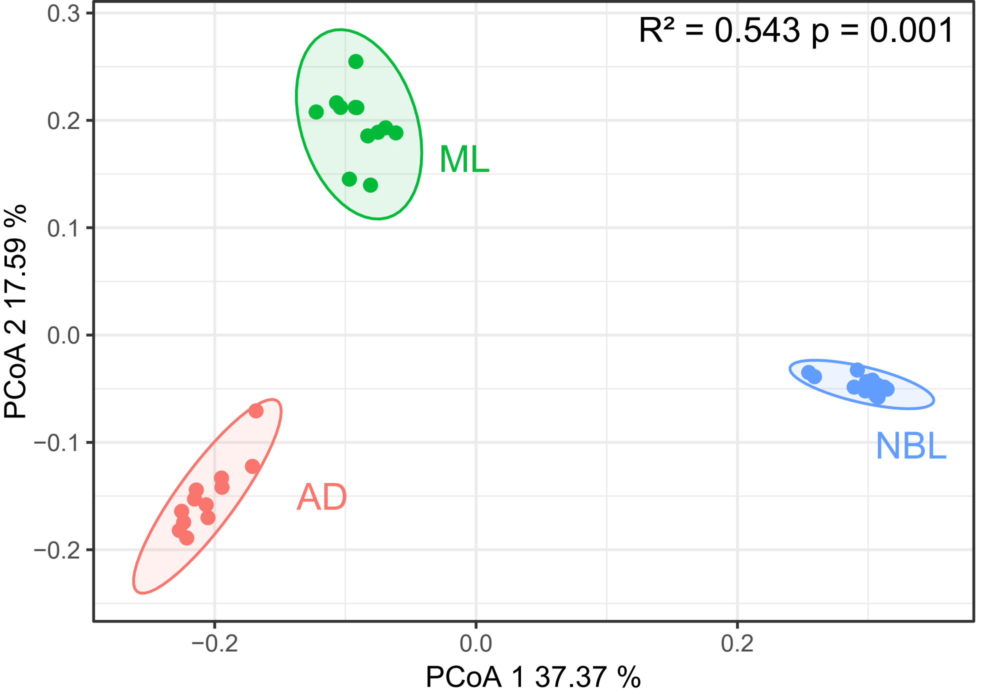

Fig. S9 Principal coordinate analysis (PCoA) of stage-specific transcriptomic profiles. The plot demonstrates significant segregation among three developmental stages based on gene expression patterns.

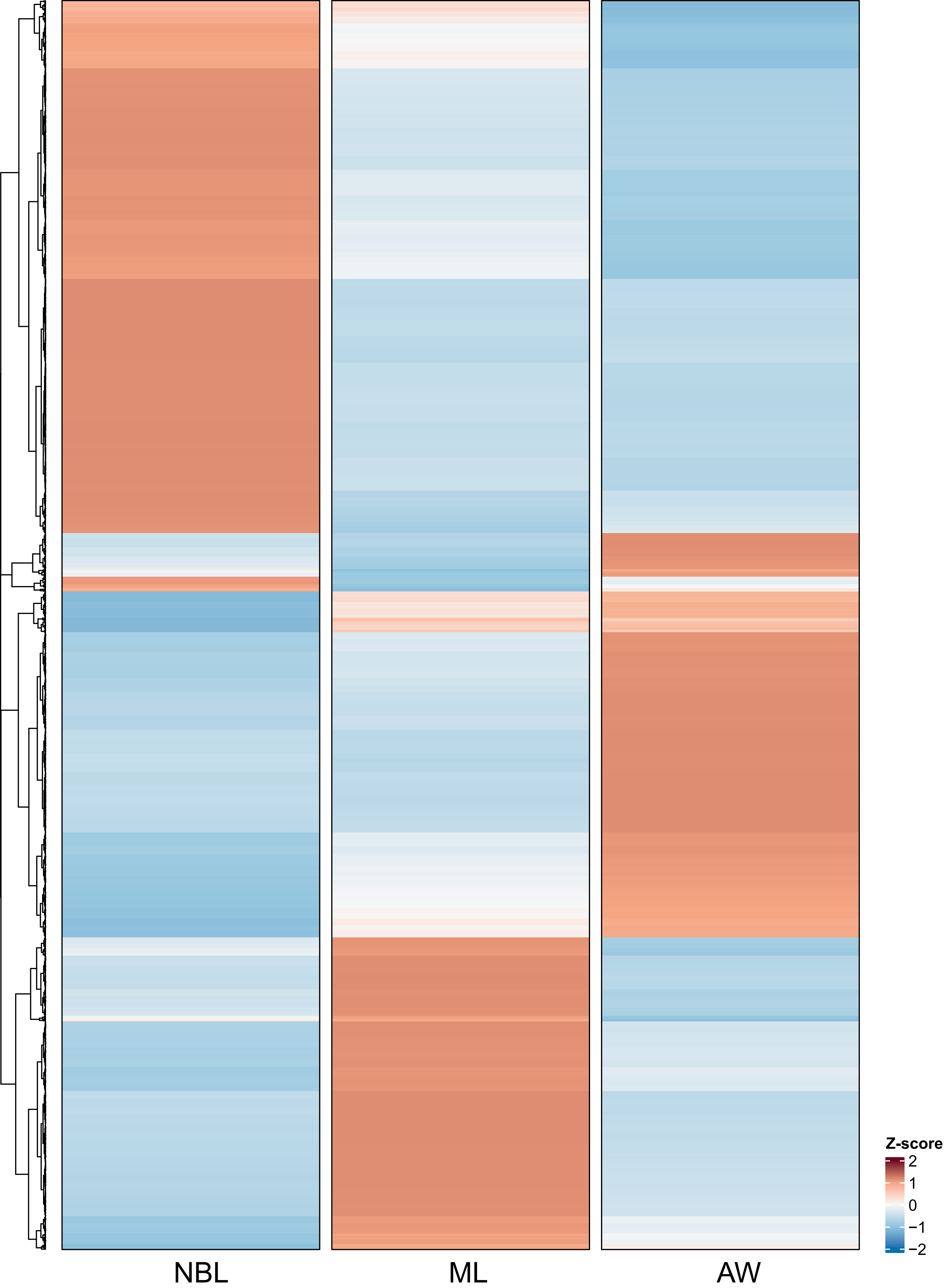

Fig. S10 Stage-specific transcriptional clusters revealed by heatmap analysis. Hierarchical clustering of gene expression profiles identifies distinct transcriptional patterns characteristic of each developmental stage.

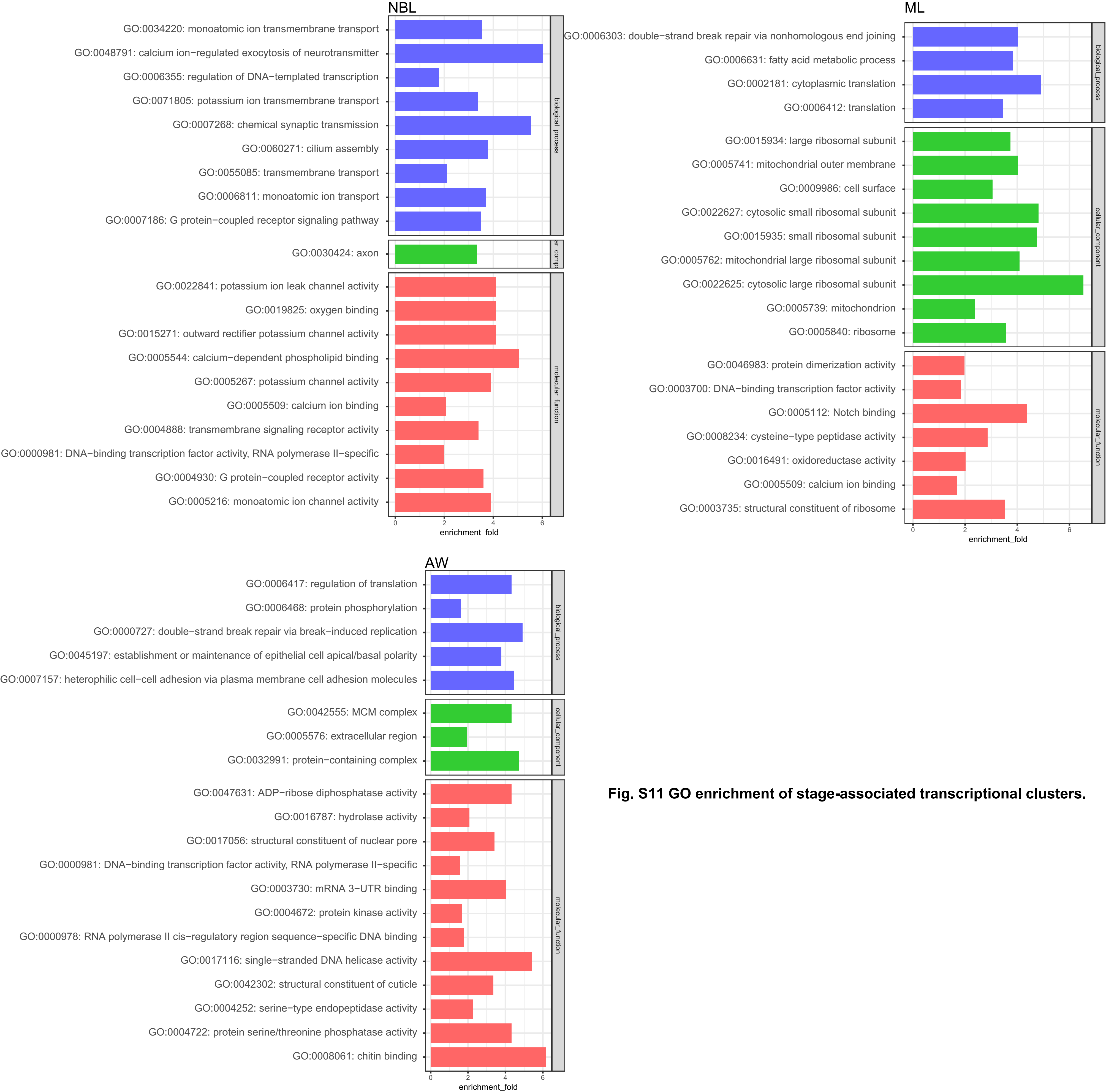

Fig. S11 GO enrichment of stage-associated transcriptional clusters.

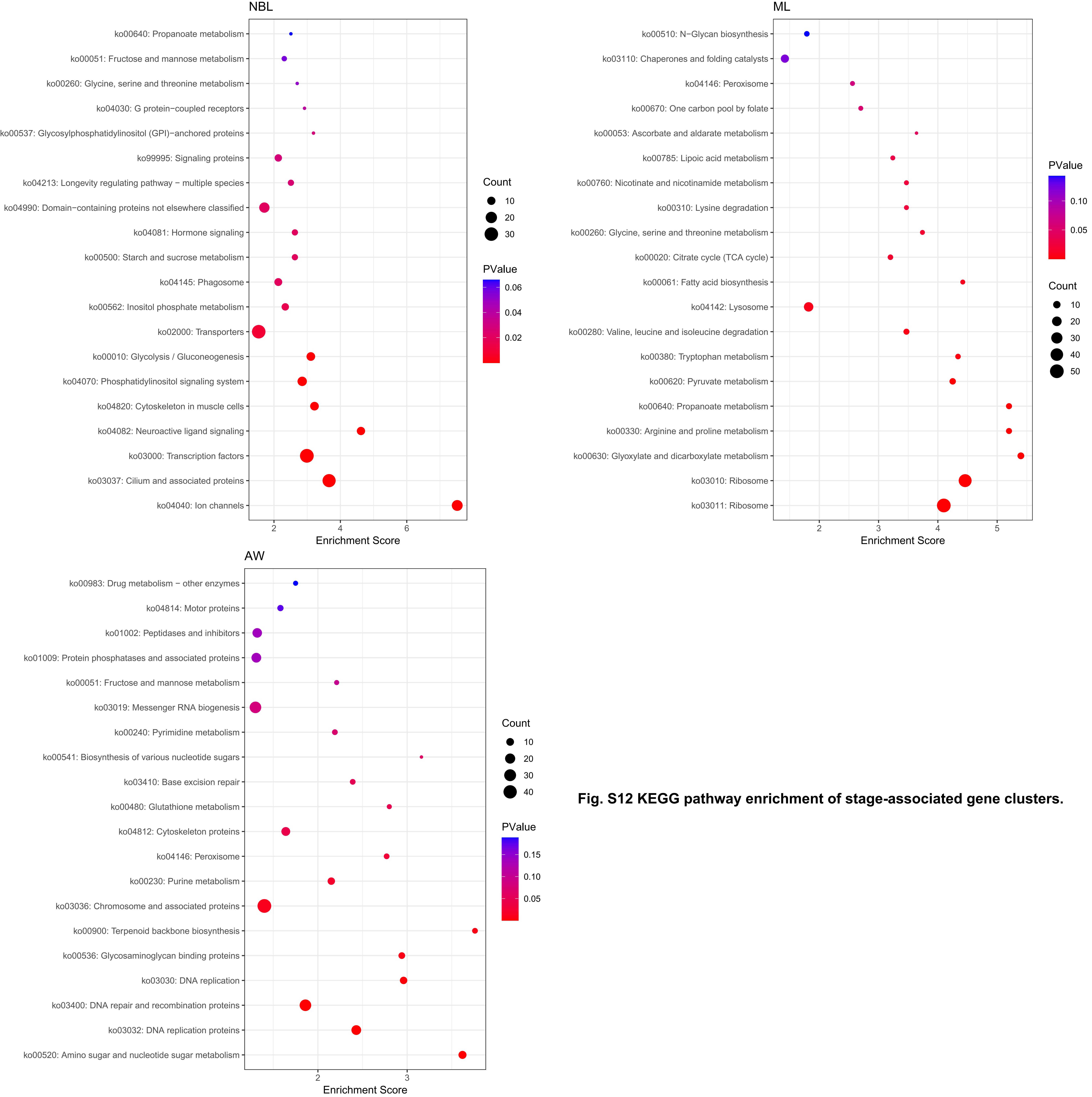

Fig. S12 KEGG pathway enrichment of stage-associated gene clusters.

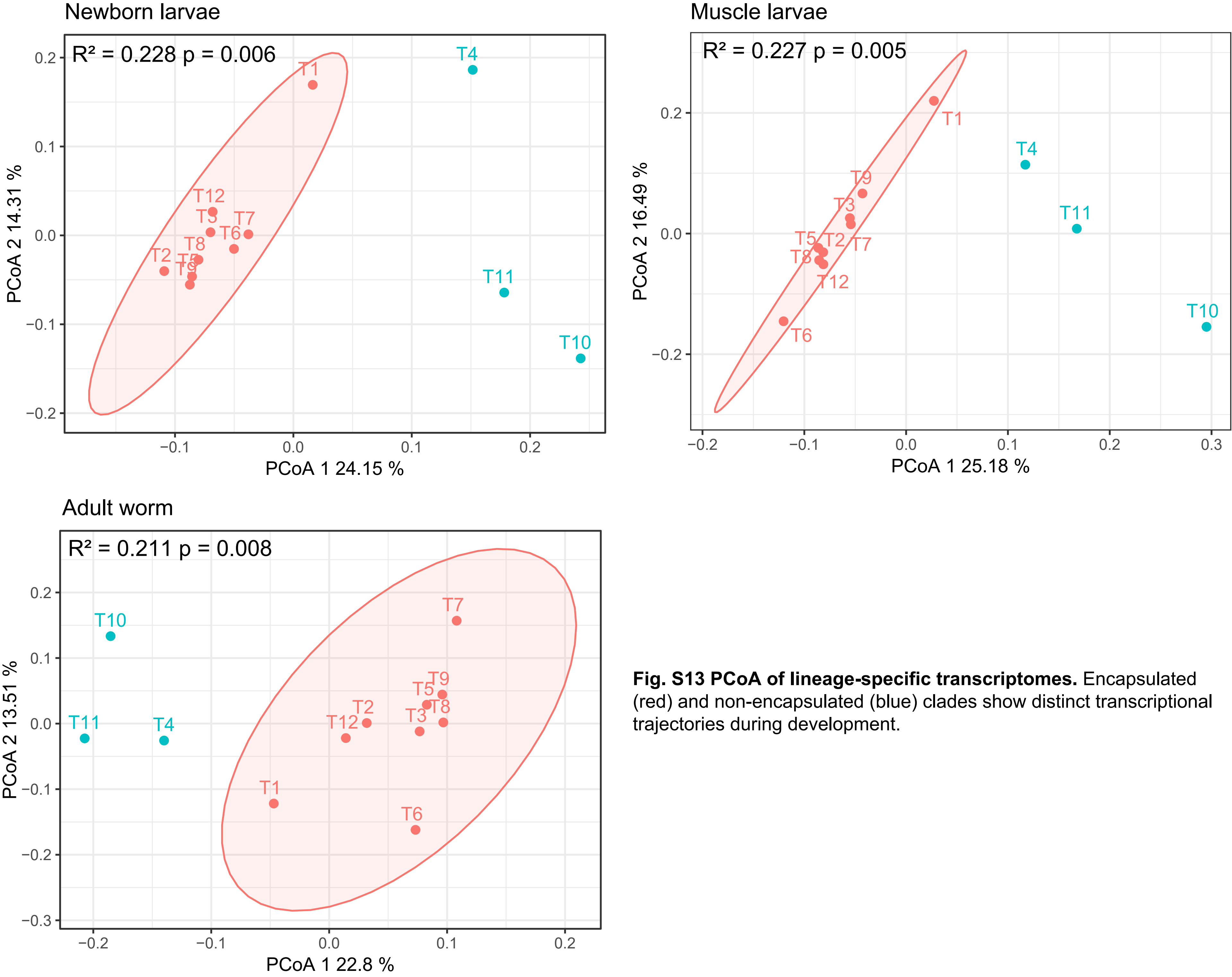

Fig. S13 PCoA of lineage-specific transcriptomes. Encapsulated (red) and non-encapsulated (blue) clades show distinct transcriptional trajectories during development.

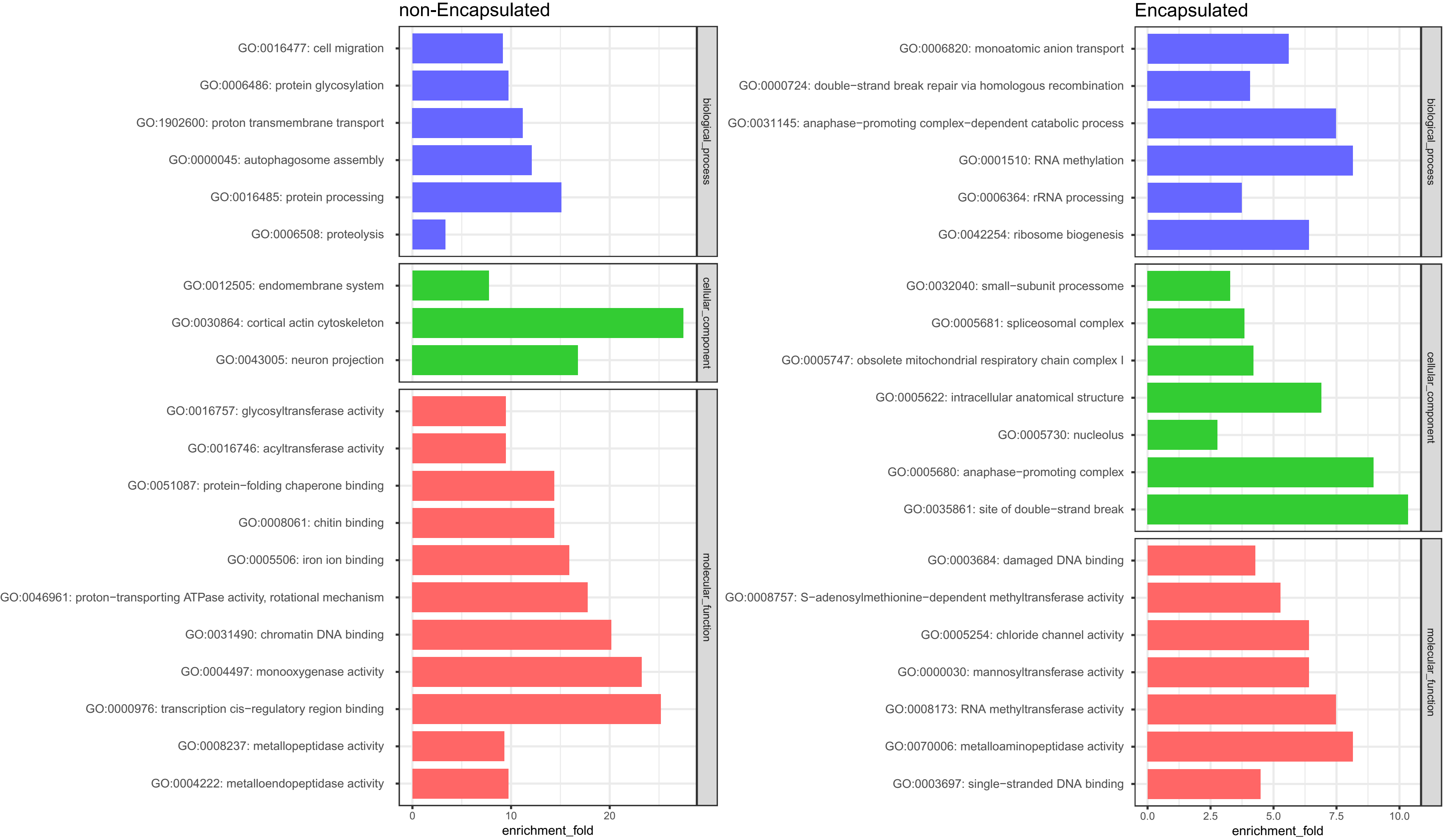

Fig. S14 GO enrichment of differentially expressed genes.

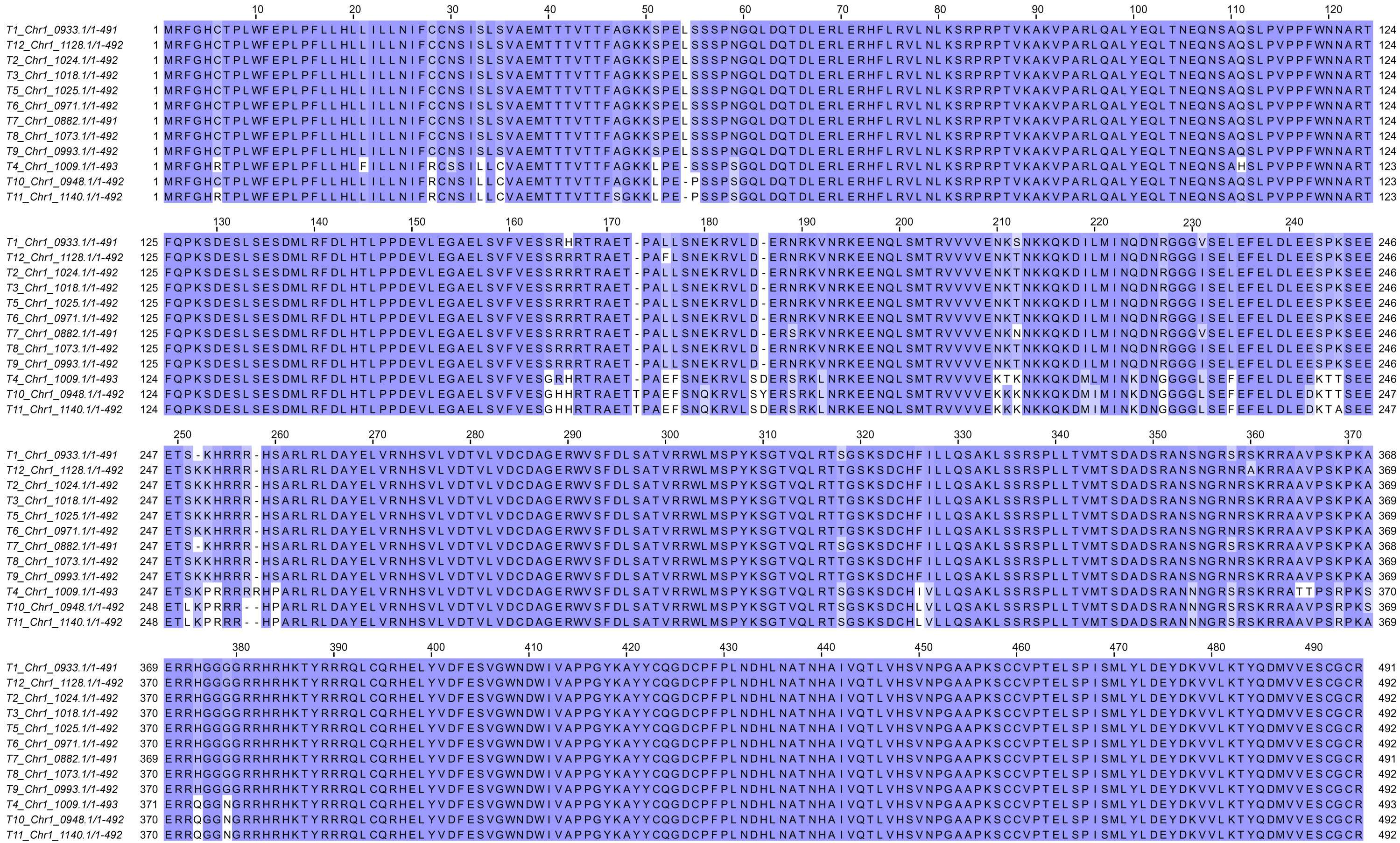

Fig. S15 *Trichinella* BMP4 amino acid sequence alignment

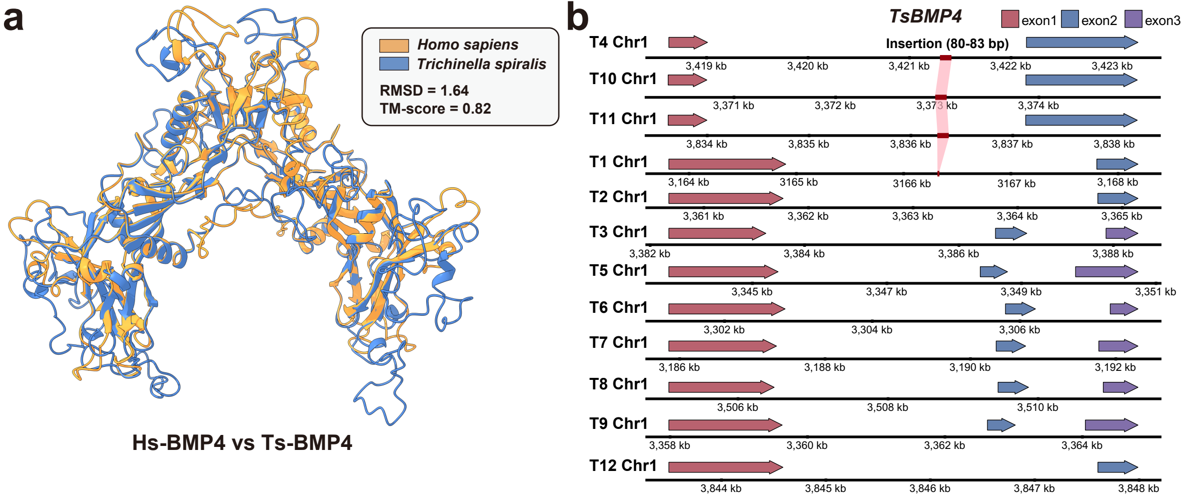

Fig. S16 Structural similarity between TsBMP4 and HsBMP4 and its structural variation in the genus *Trichinella*.
a, Pairwise structural alignment of human BMP4 and its *Trichinella* ortholog, demonstrating high three-dimensional similarity (TM-score = 0.82). **b,** Genomic alignment of the BMP4 locus across *Trichinella*.The red bar highlights an 80-83 bp intronic insertion exclusively present in all non-encapsulated lineages.
